# A Systems Biology Approach to the Analysis of Subset-Specific Responses to Lipopolysaccharide in Dendritic Cells

**DOI:** 10.1371/journal.pone.0100613

**Published:** 2014-06-20

**Authors:** David G. Hancock, Elena Shklovskaya, Thomas V. Guy, Reza Falsafi, Chris D. Fjell, William Ritchie, Robert E. W. Hancock, Barbara Fazekas de St Groth

**Affiliations:** 1 Centenary Institute of Cancer Medicine and Cell Biology, New South Wales, Australia; 2 The Discipline of Dermatology, University of Sydney, New South Wales, Australia; 3 Centre for Microbial Diseases & Immunity Research, University of British Columbia, Vancouver, British Columbia, Canada; 4 James Hogg Research Centre, University of British Columbia, St. Paul's Hospital, Vancouver, British Columbia, Canada; Karolinska Institutet, Sweden

## Abstract

Dendritic cells (DCs) are critical for regulating CD4 and CD8 T cell immunity, controlling Th1, Th2, and Th17 commitment, generating inducible Tregs, and mediating tolerance. It is believed that distinct DC subsets have evolved to control these different immune outcomes. However, how DC subsets mount different responses to inflammatory and/or tolerogenic signals in order to accomplish their divergent functions remains unclear. Lipopolysaccharide (LPS) provides an excellent model for investigating responses in closely related splenic DC subsets, as all subsets express the LPS receptor TLR4 and respond to LPS in vitro. However, previous studies of the LPS-induced DC transcriptome have been performed only on mixed DC populations. Moreover, comparisons of the in vivo response of two closely related DC subsets to LPS stimulation have not been reported in the literature to date. We compared the transcriptomes of murine splenic CD8 and CD11b DC subsets after in vivo LPS stimulation, using RNA-Seq and systems biology approaches. We identified subset-specific gene signatures, which included multiple functional immune mediators unique to each subset. To explain the observed subset-specific differences, we used a network analysis approach. While both DC subsets used a conserved set of transcription factors and major signalling pathways, the subsets showed differential regulation of sets of genes that ‘fine-tune’ the network Hubs expressed in common. We propose a model in which signalling through common pathway components is ‘fine-tuned’ by transcriptional control of subset-specific modulators, thus allowing for distinct functional outcomes in closely related DC subsets. We extend this analysis to comparable datasets from the literature and confirm that our model can account for cell subset-specific responses to LPS stimulation in multiple subpopulations in mouse and man.

## Introduction

Dendritic cells (DCs) are key regulators of T cell responses. DCs are essential for priming naive T cells and are also believed to control their effector fate. The DC lineage can be subdivided into multiple distinct subsets, some of which show intrinsic functional differences that are known to drive distinct immune outcomes [1]. However many subset-specific functional differences remain poorly understood. Here we have used a global systems approach to DC function as a means of exploring their distinct in vivo roles in the immune response.

Advances in systems biology have clearly demonstrated that linear signalling cascades poorly represent the complexity of immune signalling (reviewed in [2]). Rather than comprising linear pathways, immune signalling involves interactions between thousands of distinct proteins communicating within a complex network. These networks are organised by a set of highly connected proteins (known as Hubs) that are essential for receiving and distributing multiple signals within the network [3]–[5]. Due to their key role in the connectivity of complex signalling networks, Hubs both reflect mechanism and provide biomarkers for cell types and signalling events [3]–[5]. It is not yet known whether differences in Hub usage contribute to cell-specific differences in signalling networks.

In vivo toll-like receptor 4 (TLR4)-dependent responses to bacterial lipopolysaccharide (LPS) provide an ideal model in which to test whether closely related cell subsets show differences in their immune signalling networks, since a wide array of cell types express TLR4 and respond to LPS [6]–[9]. Studies using systems biology approaches to investigate LPS responses have primarily focused on clarifying shared mechanisms rather than defining the differences between closely related cell subsets. Published studies have shown that LPS responses are initially propagated through two sets of adaptor molecules: Toll-interleukin-1 receptor (TIR) domain-containing adaptor protein (Tirap) and Myeloid differentiation primary response 88 (Myd88) (Tirap-Myd88, the ‘Myd88-dependent pathway’), or TIR-domain-containing adapter interferon-β-inducing factor (Trif) and TRIF-related adaptor molecule (Tram) (Trif-Tram, the ‘Myd88-independent pathway’). Additionally, a set of Hubs responsible for orchestrating signalling outcomes in response to LPS has been defined [6]–[9]. These Hubs are essential for signal propagation and belong primarily to the tumor necrosis factor receptor associated factor (TRAF), interleukin-1 receptor-associated kinase (IRAK), mitogen-activated protein kinase (MAPK) and nuclear factor kappa-light-chain-enhancer of activated B cells (NFκB) families (extensively reviewed in [6]–[9]).

Transcriptional analysis of the LPS response in murine DCs has generally been confined to cells differentiated in vitro from bone marrow precursors, with a single report of the response of unfractionated ex vivo splenic DCs [10]–[15]. The splenic DC compartment comprises distinct cell subsets expressing either CD8 or CD11b and manifesting different basal transcriptional programs [16]–[26]. CD8 DCs are thought to uniquely cross-present antigen to CD8 T cells and are the major producers of interleukin (IL-12) for the regulation of Th1 responses, while CD11b DCs are thought to be dominant in the regulation of CD4 T cell responses and Th2 immunity [1], [24], [27], although not all models support these functional distinctions [28]. Inactivation of key transcription factors, including IRF8, BATF3, IRF4 and Ikaros, selectively interferes with development of CD8 or CD11b DC subsets [29], [30]. However differences in inflammatory signalling pathways in the two subsets remain poorly defined. Both have been reported to respond directly to LPS in vitro and in vivo, although they express relatively low basal levels of TLR4 [31], [32]. In vitro stimulation with LPS induces equivalent production of tumor necrosis factor alpha (TNFα) and IL-6, but higher production of IL-12 in CD8 DCs, suggesting that while both subsets share common signalling pathways and TLR4 potency, subset-specific differences are also present [31]. Both DC subsets also respond to a number of mediators released by DCs and other cell types in response to LPS, so that their response to in vivo LPS administration comprises a network of direct and indirect effects that jointly control their ability to differentially stimulate T cells [1], [27]. Such effects may not be adequately modelled by in vitro LPS stimulation of purified cells.

In this study, we administered LPS in vivo and isolated splenic DCs from untreated and LPS-treated mice with minimal manipulation. We then used RNA-Seq of flow-sorted samples pooled from multiple mice to compare the physiological responses of closely related DC subsets to in vivo LPS exposure. Using a hyper-stringent method for choosing differentially expressed genes in the DESeq R package [33], we show that CD8 and CD11b splenic DC subsets respond differently to in vivo LPS stimulation, and that many of the transcriptional changes previously defined in the LPS response of unfractionated DCs are present in only one of the two subsets.

We used network analysis to identify a subnetwork in each DC type in order to elucidate the mechanisms underlying the observed differences. Such subnetworks are thought to contain key regulators of the measured response. Importantly, they are self-reinforcing, and are therefore less susceptible to variability in individual gene detection [3]–[5]. The 2 DC subsets generally expressed the same set of core LPS response molecules, many of which served as Hubs in each subset-specific subnetwork. Both subsets also expressed a common set of cell-surface receptors required for responses to secondary mediators released after LPS stimulation. However, the sets of proteins interacting with these core Hubs were significantly different in the 2 subsets. Importantly, the majority of such interacting proteins, including Atf3, Tnfaip3 (A20), Tradd and Cdkn1a, are already known to be modulators of common signalling pathways, although they had not previously been accorded subset-specific roles. These data support a model in which distinct immune responses to the same stimulus are achieved by differential ‘fine-tuning’ of core pathways by subset-specific modulators. Finally, we validated our hypothesised model using meta-analyses of other cell populations, showing its relevance to inflammatory LPS signalling in multiple cell subsets in mouse and human.

## Results

### Differential activation of DC subsets by in vivo exposure to LPS

**Figure 1:**
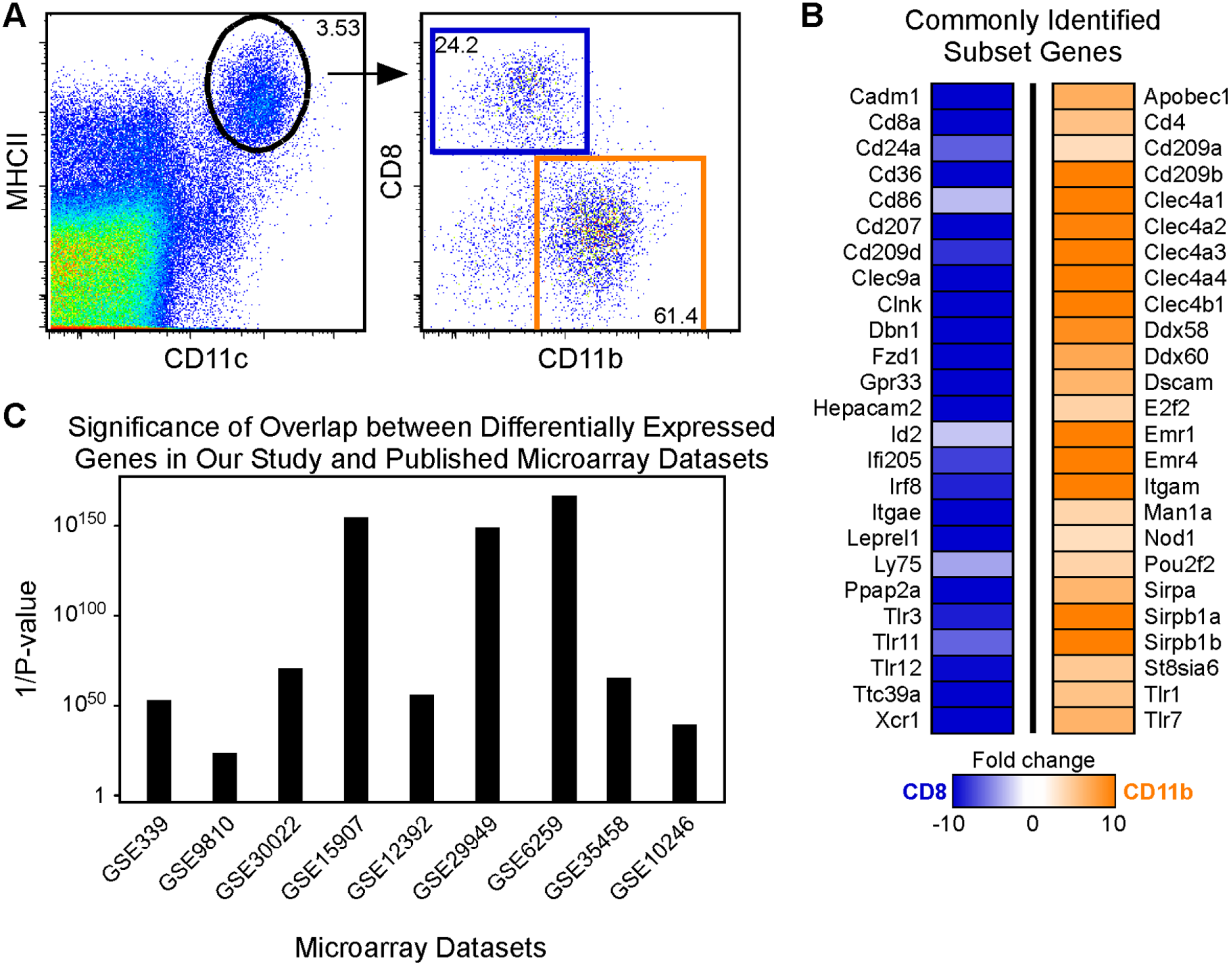
Comparison of steady-state spleen DC subsets. (A) Steady-state splenic DCs were magnetic bead-enriched for CD19^−^B220^−^CD3^−^Gr-1^−^Ter119^−^CD11c^+^ cells. MHCII^+^CD11c^+^ cells (circled) were then sorted for CD8 (blue gate) and CD11b (orange gate) subsets and RNA prepared and analysed by RNA-Seq. (B) Heatmap showing the relative expression of the 50 most commonly defined and validated markers for CD8 and CD11b subsets. Data are presented as fold changes (CD11b/CD8), all of which were statistically significant with an associated p-value <0.05. Orange denotes genes that were increased in CD11b DCs while blue denotes genes that were decreased in CD11b DCs (and thus increased in CD8 DCs. (C) Overlap between genes that were significantly differentially expressed between CD8 and CD11b DCs in our dataset and in 9 previously published microarray datasets derived from splenic DC subsets (datasets 1–9 listed in Table S1). The significance of overlap between the gene list from our dataset and those from each of the published datasets was calculated using a hypergeometric test to assess the consistency/quality of our results. Data are presented as 1/p-value on a log scale with all overlaps reaching a significance cut-off <0.05.

**Figure 2:**
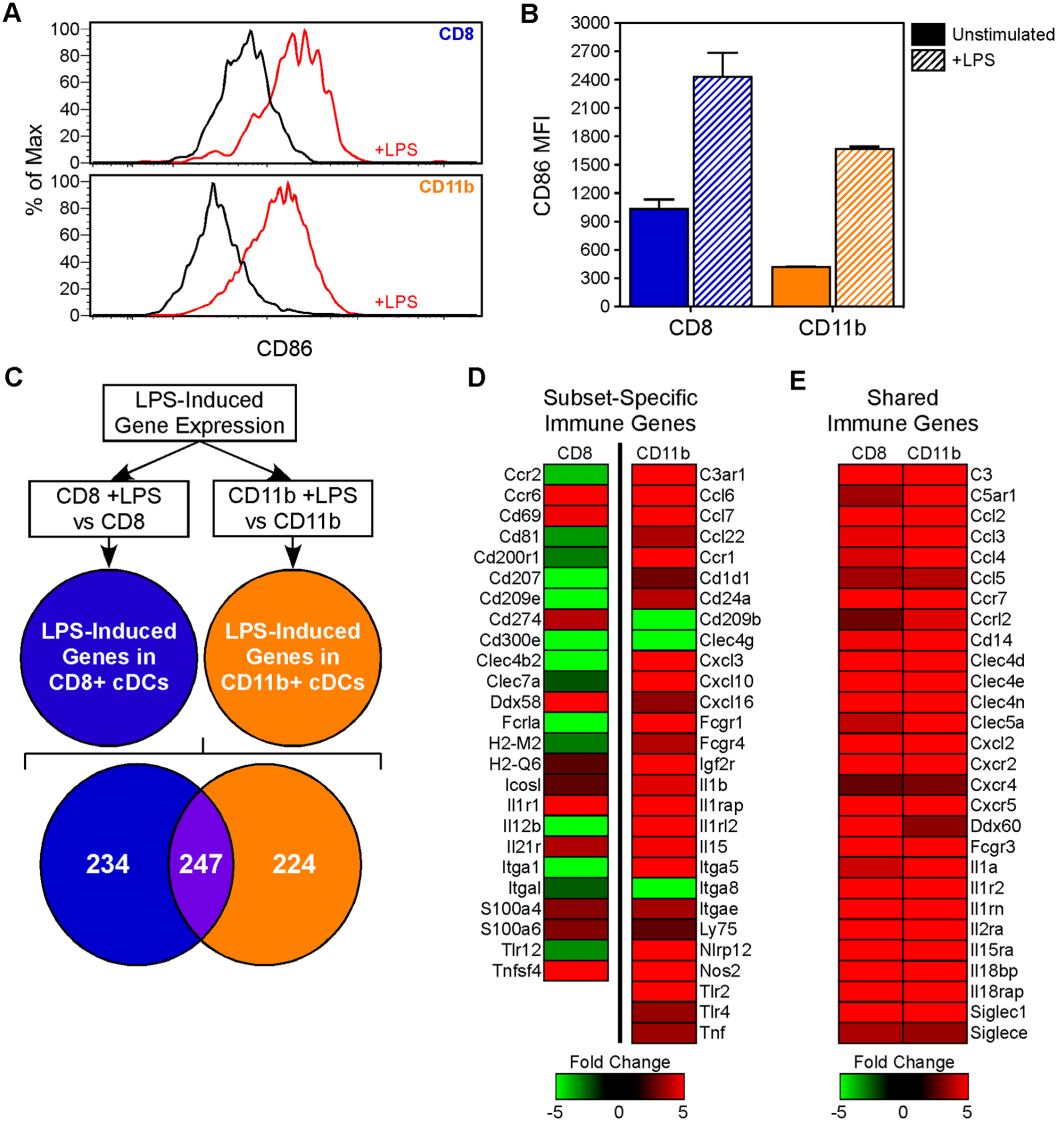
Subset-specific LPS-induced gene signatures. (A–B) Dendritic cells were stimulated in vivo with an intraperitoneal injection of 25ug LPS and their activation confirmed by measuring upregulation of CD86 24 hours later. (C–E) LPS stimulated DC subsets isolated from a second cohort of mice (n = 10, spleen cells pooled) were magnetic bead enriched, sorted, analysed by RNA-Seq, and then compared to steady-state controls (n = 5, spleen cells pooled). (C) Differences in LPS-induced gene expression were visualised in a Venn diagram. (D–E) Heatmaps showing the expression of key immune effector genes (D) uniquely regulated in either CD8 (left) or CD11b DCs (right) or (E) similarly regulated in both subsets. Data are presented as fold changes (+LPS/−LPS).

Spleen cells were harvested from steady-state (n = 5) and LPS-treated (n = 10, 24 hours after 25 μg LPS i.p) mice and DC subsets purified using magnetic bead enrichment for lineage (CD19, B220, CD3, Gr-1, Ter119)-negative, CD11c-positive cells, followed by flow sorting according to the gating strategy shown in Figure 1A. We confirmed that DCs were indeed activated by in vivo LPS administration by comparing expression of the activation marker CD86 to that of steady-state cells (Figure 2A–B). As expected [34], both DC subsets responded to LPS in vivo by up-regulating CD86. We also confirmed that our enrichment and gating strategy for steady-state and LPS-treated DCs excluded monocyte-derived DCs identified on the basis of coexpression of FcγR1 (CD64) and FcεR1α [35] (Figure S1). RNA-Seq was then performed on the 4 RNA samples, using standard techniques as described in the Materials and Methods section. Significantly differentially expressed genes were identified using a hyper-stringent method from the R package DESeq at a p-value cut-off <0.05 [33]. We assessed the quality of our RNA-Seq data by comparing our steady-state data with published results from the literature. We first compiled a set of the top 50 prototypical subset-specific genes, based on published studies of mRNA and protein expression, and showed that our steady-state data faithfully recapitulated the published patterns (Figure 1B). Next we calculated the overlap between our steady-state data and 9 published datasets (datasets 1–9 listed in Table S1 [16]–[26]) using a hypergeometric test (Figure 1C, see Materials and Methods). The highly significant overlap between differentially expressed genes in our steady-state dataset and each of the published datasets indicated that our steady-state data were remarkably consistent with previously published data. These analyses indicated that our dataset provided a suitable measure of gene expression, even though it contained only a single pooled sample for each experimental condition.

We next analysed LPS-induced gene expression by comparing LPS stimulated with steady-state data for each subset. LPS stimulation of CD8 DCs led to a significant change in the expression of 481 genes (397 upregulated, 84 downregulated), while in CD11b DCs there was a significant change in the expression of 471 genes (428 upregulated, 43 downregulated) (Table S3). We also confirmed that both subsets expressed detectable Tlr4 mRNA (CD8 DCs: 59 and CD11b DCs: 92 normalised counts per million). Interestingly, the observed LPS responses were highly subset-specific. Thus 49% of differentially regulated transcripts in CD8 DCs were subset-specific, while the corresponding figure for CD11b DCs was 48% (Figure 2C). Many of these differentially regulated genes are known to be important immune effector genes, suggesting an LPS-regulated functional divergence between the 2 DC subsets. Compared with CD8 DCs, CD11b DCs selectively upregulated a wider range of transcripts encoding cytokines/chemokines (including Ccl6, Ccl7, Ccl22, Cxcl3, Cxcl10, Cxcl16, Il1b, Il15, and Tnf), while CD8 DCs selectively upregulated co-stimulatory molecules such as Cd274 (PD-L1), Icosl, and Tnfsf4 (OX40L) (Figure 2D). These transcriptional changes are likely to be mediated by a combination of direct (LPS-TLR4) signals and secondary soluble (cytokines/chemokines) and/or cell-to-cell signals from the LPS-activated splenic microenvironment.

To identify known biological pathways underlying the differences between the subsets, we performed gene ontology (GO) term over-representation analysis on the significantly differentially regulated genes. The GO term analysis afforded further evidence that our data from single pooled samples provided a valid measure of gene expression. Thus genes annotated by GO terms associated with LPS-stimulation (response to lipopolysaccharide and cellular response to lipopolysaccharide) and with the general inflammatory response (cytokine-mediated signalling pathway, immune response, inflammatory response, innate immune response) were significantly enriched in both subsets, although the individual genes within the GO categories differed between the subsets (Table 1). GO terms associated with regulation of the apoptotic process were enriched in both subsets although they reached statistical significance only in CD11b DCs, while the GO term ‘negative regulation of the inflammatory response’ was significantly enriched only within the CD8 DC subset (Table 1). Interestingly both apoptosis and negative regulation are characteristic of the so-called “late” LPS response [6]–[9].

**Table 1:** GO terms annotating LPS-induced genes in CD8 versus CD11b DCs.

GO Term	CD8 DCs		CD11b DCs	
	Pval^2^	Odds-Ratio^1^	Pval^2^	Odds-Ratio^1^
Aging	1.9e-03	4.7	2.7e-04	5.2
cell adhesion	8.7e-03	3.4	1.0e-03	3.7
cellular response to lipopolysaccharide	2.7e-08	13.3	9.0e-10	15.1
cytokine-mediated signalling pathway	1.8e-04	7.7	5.4e-04	7.1
G-protein coupled receptor signalling pathway	7.3e-05	3.0	3.0e-05	3.1
immune response	7.0e-08	5.4	2.4e-13	7.8
inflammatory response	3.1e-08	5.7	7.3e-15	8.5
innate immune response	2.8e-04	4.9	2.7e-08	7.1
positive regulation of angiogenesis	8.7e-05	9.7	5.4e-06	11.2
response to drug	9.8e-04	3.2	4.7e-10	5.1
response to estradiol stimulus	9.2e-03	5.7	4.5e-03	5.9
response to lipopolysaccharide	2.8e-10	7.8	4.8e-19	12.3
response to organic cyclic compound	3.4e-02	3.8	3.4e-04	5.1
response to virus	4.2e-06	6.8	1.2e-05	6.4
anti-apoptosis	3.3e-01*	2.7	1.4e-02	3.7
negative regulation of apoptotic process	4.6e-01*	2.0	3.4e-03	2.9
positive regulation of apoptotic process	1.5e-01*	2.6	1.7e-03	3.5
proteolysis	4.7e-01*	1.9	4.6e-02	2.4
negative regulation of inflammatory response	6.7e-05	12.0	1.9e-01*	4.9
positive regulation of gene expression	5.7e-03	4.3	6.3e-02*	3.3

GO term over-representation analysis of LPS-induced genes in CD8 and CD11b DCs.

1. The ratio of odds (Odds-Ratio) that a GO term is enriched in the selected DC subset was calculated as the odds of a differentially expressed gene divided by the odds of a non-differentially expressed gene occurring in the GO term.

2. P-values are adjusted to control for multiple comparisons.

*denotes not significant (p>0.05).

### Comparison of DC subset-specific LPS responses with published analyses of unfractionated DC responses

In contrast to the extensive published microarray characterisation of steady state DC subsets, most studies assessing LPS responses in DCs have used in vitro-derived DCs. We compared our data with these LPS response datasets to test how many of the significantly differentially expressed genes specific for each subset had also been identified within the published datasets. DC responses to LPS have usually been modelled using murine bone-marrow (BM)-DCs derived from in vitro stimulation of BM cells matured into DCs in 5–8 day cultures containing granulocyte-macrophage colony-stimulating factor (GM-CSF), alone or in combination with IL-4, IL-3, IL-6, and/or stem cell factor (SCF) [36], [37]. While BM-DCs can be subdivided into CD24- and CD11b-expressing subpopulations [36]–[38], they have been analysed by microarray only as a ‘mixed’ population. LPS responses are dynamically regulated over time and influenced by multiple factors including the type and dose of LPS [13], [31], [39]. We reanalysed 5 published microarray datasets, containing a total of 10 timepoints (datasets 10–14 listed in Table S1, [10]–[14]), and identified 12,886 LPS responsive genes (p-value <0.05) in BM-DCs.

**Figure 3:**
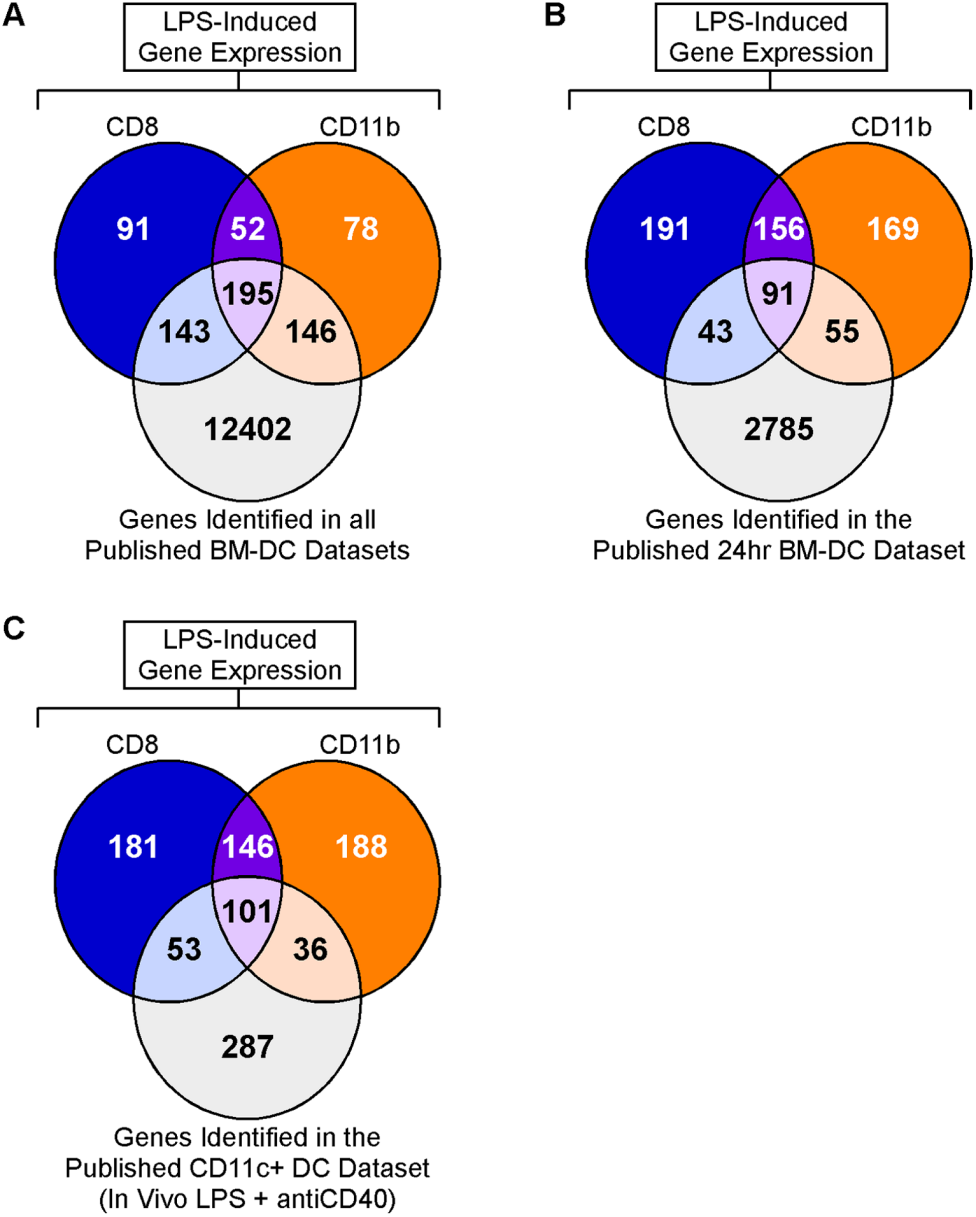
Population vs. subset-specific LPS-responses. (A) Data from published studies comparing in vitro stimulated GM-CSF derived BM-DCs +/− LPS stimulation (datasets 10–14 listed in Table S1) were reanalysed for differential gene expression. All LPS-induced genes identified in BM-DCs at any of the 10 timepoints (p-value <0.05) were compared to LPS-induced genes in our study and visualised in a Venn diagram. (B) Only genes differentially expressed in the 24 hour timepoint sample from the GSE17721 BM-DC dataset (dataset 13 listed in Table S1) were compared to LPS-induced genes in our study and visualised in a Venn diagram. (C) Genes identified as significantly differentially expressed (p-value <0.05) in splenic CD11c^+^ DCs stimulated in vivo with LPS and anti-CD40 and isolated after 6 hours were compared to in vivo LPS-induced genes in our study (isolated after 24 hours) and visualised in a Venn diagram (dataset 15 listed in Table S1).

We then performed 2 different analyses comparing our 24 hour timepoint data from sorted DC subsets with the published datasets. In the first, we included all 12,886 published LPS-responsive genes in the comparison. Of the 705 LPS responsive genes identified in our in vivo studies, 484 (69%) were also identified in at least one of the in vitro BM-DC datasets (Figure 3A). The high degree of overlap between our in vivo activated DCs and in vitro stimulated BM-DCs further validates the quality of our results and suggests that a major part of the observed transcriptional response in our ex vivo DCs was directly TLR4-mediated. However, 289 (60%) of these 484 commonly identified genes were regulated in a subset-specific manner in either CD8 (143 genes) or CD11b (146 genes) DCs after in vivo LPS stimulation (Figure 3A). Next we repeated our comparison using only the 24 hour timepoint from the GSE17721 dataset [13], the sample that was the most consistent with our experimental setup. A similar trend was observed in this analysis, with 98 (52%) of the 189 genes identified in both studies being regulated in a subset-specific manner in our dataset (Figure 3B).

We also reanalysed the only published microarray dataset derived from splenic DCs isolated directly from LPS-treated mice (dataset 15 listed in Table S1, [15]). These cells were stimulated for 6 hours in vivo with a combination of LPS and anti-CD40, and isolated on the basis of CD11c expression without fractionation into subsets [15]. Despite differences in stimulus and timepoint, 190 of our 705 genes were also identified in this dataset (Figure 3C). Consistent with our observed subset specificity, 47% of the 190 were differentially regulated between subsets in our RNA-Seq analysis (Figure 3C).

These results indicate a degree of DC subset-specificity within the LPS response that had not previously been apparent from analysis of unfractionated populations such as BM-DCs and CD11c-expressing splenic DCs. Many of the previously identified ‘LPS-responsive genes’ may be regulated within only a subset of the total population, and novel subset-specific genes may be missed in such analyses.

### Splenic DC subsets share a common set of LPS-responsive transcription factors and signalling molecules

Subset-specific transcriptional responses to LPS have been reported in non-DC cell types, but the mechanisms underlying this specificity have not been thoroughly addressed [40], [41]. To identify molecular mechanisms underlying the observed transcriptional differences between the 2 closely related splenic DC subsets in our study, we employed a number of systems biology approaches. As a first step, we tested whether there were major primary signalling differences between the 2 subsets by looking for differential expression and activity of key LPS-response molecules, including transcription factors.

We performed over-representation analysis on transcription factor-target LPS-induced genes in each subset, using a list of transcription factor-gene interactions based on experimental evidence and downloaded from innateDB [42]. Both subsets were significantly enriched for genes regulated by the transcription factors Cebpb, Irf1, Irf8, Jun, Nfkb1, Rela, and Sp1, although the individual genesets were only partially overlapping between the subsets (Table 2). Genes regulated by the transcription factor Egr1 were enriched in both subsets, although the effect narrowly failed to reach statistical significance in CD8 DCs (p-value 0.062). Importantly, all these transcription factors were constitutively expressed by both DC subsets and are known to play key roles in LPS responses [6]–[9].

**Table 2:** Expression of canonical transcription factor-target TLR4-dependent pathways mediating the LPS response in CD8 and CD11b DCs.

Transcription Factor	CD8 DCs		CD11b DCs	
	Pval^2^	Odds-Ratio^1^	Pval^2^	Odds-Ratio^1^
Cebpb	3.6e-05	6.2	2.8e-06	6.2
Irf1	4.6e-08	7.4	8.1e-12	8.4
Irf8	4.3e-06	2.6	9.6e-13	3.3
Jun	9.6e-06	7.5	2.7e-10	11.2
Nfkb1	4.3e-06	6.5	1.2e-11	9.7
Rela	2.2e-11	7.9	9.6e-13	7.3
Sp1	9.6e-06	8.2	1.1e-02	3.5
Egr1	6.2e-02*	1.3	5.1e-03	1.3

Transcription factor-target over-representation analysis of LPS-induced genes in CD8 and CD11b DCs.

1. The ratio of odds (Odds-Ratio) that a transcription factor-associated pathway is enriched in the selected DC subset was calculated as the odds of differentially expressed genes being regulated by the transcription factor divided by the odds of non-differentially expressed genes being regulated by the same transcription factor.

2. P-values are adjusted to control for multiple comparisons.

*denotes not significant (p>0.05).

We also compared the relative expression of core LPS response molecules in the steady-state and after LPS stimulation. 48 core molecules were curated either as key canonical signalling molecules in LPS responses defined in multiple publications [6]–[9] and/or identified within the KEGG and Reactome databases (Figure S2). Importantly, these core molecules are also known to serve as essential mediators of many other immune signalling pathways and thus would be predicted to function as Hubs in both primary LPS-TLR4 and secondary signalling pathways. Of the 48 core molecules, only 3 (Ticam2, Tlr4 and Ikbke) showed differential expression between the 2 subsets, and none were consistently differentially expressed both before and after LPS stimulation (Figure S2). The TLR4 signalling adaptor protein, Ticam2 (Trif) was significantly upregulated in CD11b DCs only before stimulation, whereas Tlr4 itself and the signalling molecule, Ikbke, were significantly upregulated in the CD11b subset only after stimulation (Figure S2).

In this analysis, clear subset-specific differences in key transcription factors and core signalling molecules could not be identified. Instead, these data support a model in which both DC subsets signal through a common set of molecules.

### DC subset-specific responses are ‘fine-tuned’ by distinct pathway modulators

**Figure 4:**
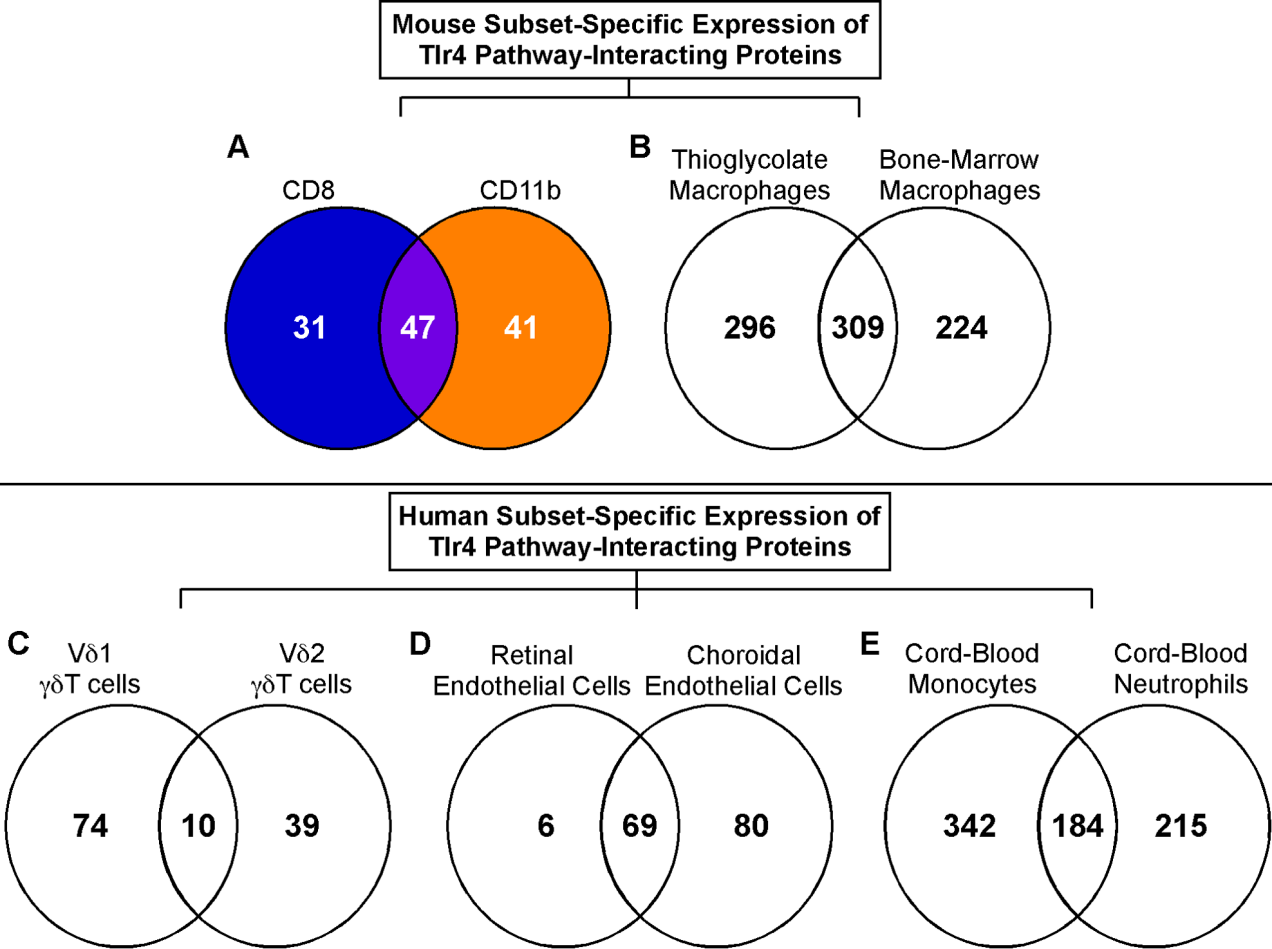
Subset-specific expression of pathway modulators. LPS-induced regulation (+LPS/−LPS) of pathway modulators in mouse (A–B) and human (C–D) cell subsets, visualised in Venn diagrams. (A) CD8 and CD11b DCs from our study. (B) Thioglycolate-elicited peritoneal macrophages and bone-marrow derived macrophages [26]. (C) Vδ1 and Vδ2 γδ T cells [40]. (D) Retinal vascular endothelium and choroidal endothelial cells [41]. (E) Cord blood peripheral blood monocytes and neutrophils [48]. Datasets in (B–E) are listed as 16–19 in Table S1.

We next performed network analysis on the differentially expressed genes in each subset, in order to identify potential modulators of the subset-specific responses. As a first step, we uploaded our defined list of 48 core LPS response molecules into the immune database and analysis platform InnateDB, to generate a network containing these molecules and their first-order interacting partners. This identified 2279 interacting genes. We then filtered this network for interacting nodes (genes) that were significantly differentially expressed in the DC subsets. This filtered analysis identified multiple differences in LPS-responsive genes that interact with, and are known to modulate, the function of the 48 core signalling molecules (Figure 4A, Table S4). CD8 DCs uniquely regulated Anxa2, Atf3, Birc2, Cd81, Ctnnd1, Ddx58, Dnajb1, Egr1, Fkbp5, Gadd45g, Hspa1b, Ikzf4, Il12b, Il1r1, Itgam, Jak3, Ksr1, Map2k6, Mef2c, Nfkbia, Peli2, Prdm1, Pygl, Relb, Sertad1, Spib, Stat4, Tgm2, Tnfaip3, Traf1, and Zfp36. CD11b DCs uniquely regulated Atxn1, Bcl2l1, Bcl2l11, Cd209b, Cdkn1a, Erc1, Fosl2, Fth1, Gadd45a, Hmox1, Id1, Id2, Igf2r, Il15, Il1rap, Jdp2, Map4k4, Myo1d, Nfe2, Nlrp12, Nos2, Notch1, Notch3, Optn, Pld3, Plin2, Pnrc1, Prdx5, Rhbdf2, Rhoc, Sh3bp5, Slc12a2, Snap23, Sod2, Tlr2, Tlr4, Tnf, Trib1, Trim29, Tuba8, and Usp2.

**Figure 5:**
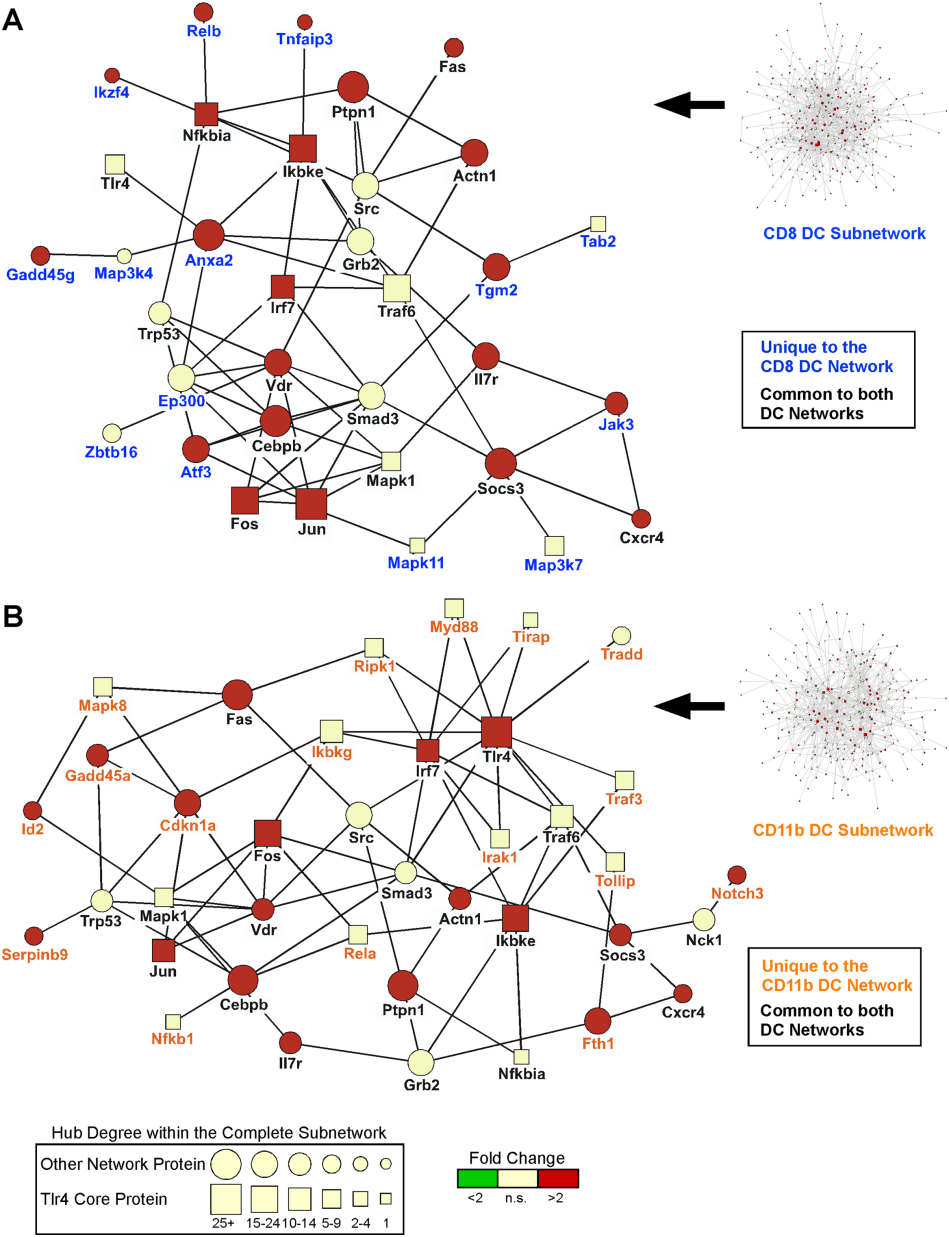
Network analysis of LPS-responsive genes in CD8 and CD11b DCs. Individual network analyses were carried out on the transcriptional response of (A) CD8 and (B) CD11b DCs stimulated in vivo with LPS as compared to steady-state. Subnetwork analysis was used to enrich networks in an unbiased manner for interactions with differentially expressed genes. The full subnetworks for each subset (Right Panels) were edited to show a smaller core network for ease of visualisation while retaining the original topology. These smaller core networks include those genes with the largest Hub degree (interconnectivity with other genes in the full subnetwork), all core LPS response molecules, and key subset-specific modulators interacting with these genes. Subset-specific nodes are labelled in blue (unique to the CD8 subnetwork) or orange (unique to the CD11b subnetwork), while nodes labelled in black text are present in both subnetworks. The size of each node is proportional to its Hub degree, while node colour indicates relative gene expression (+LPS/−LPS). Square nodes represent core LPS response molecules. The full subnetwork diagrams, made using the Cytoscape plugin Cerebral to show the cellular localisation of each gene, are displayed in Figures S3 and S4, while a list of nodes/network characteristics is provided in Tables S5 and S6.

A limitation of this method for finding differentially regulated pathway modulators is that their identification was based on known interactions with a predefined list of core signalling molecules involved in LPS responses. As an alternate unbiased approach, we generated first-order interaction subset-specific networks from all differentially expressed genes in each subset, and then applied an unbiased subnetwork analysis. This successfully yielded core subnetworks containing 317 individual nodes for CD8 DCs and 297 nodes for CD11b DCs (Figure 5, Figures S3–S4, Tables S5–S6). These unbiased subnetworks, which include the most interconnected genes involved in the LPS response of each DC subset, demonstrated less than 50% overlap with each other, indicating significant differences in response between the 2 subsets. To initially characterise the 2 subnetworks, Pathway (from Reactome) and GO term over-representation analysis was performed on the genes comprising the subnetworks. No significant differences in Reactome Pathways were observed (Table 3). Both subnetworks were significantly enriched in nodes annotated by Reactome as being in pathways relating to TLR4 signalling, including both Myd88-dependent and -independent cascades (Table 3). GO terms relating to the LPS-response, the MAPK and NFkB cascades, and the general inflammatory responses were also over-represented in both networks (Table S2).

**Table 3:** Reactome pathways enriched within the CD8 and CD11b DC subnetworks.

Reactome Pathway	CD8 DCs	CD11b DCs
	Pval	Pval
Activated TLR4 signalling	1.2e-06	7.3e-11
Adaptive Immune System	1.7e-04	3.1e-09
Cell-Cell communication	2.5e-05	4.4e-03
Cytokine signaling in Immune system	5.1e-21	2.4e-19
Growth hormone receptor signaling	3.6e-10	1.6e-10
Immune System	2.9e-26	4.6e-29
Innate Immune System	1.5e-11	8.0e-16
Integrin cell surface interactions	3.0e-09	1.4e-08
Interferon signaling	8.3e-11	3.4e-05
Interleukin-2 signaling	1.8e-06	7.3e-06
Interleukin-3, 5 and GM-CSF signaling	2.6e-07	8.9e-06
MyD88 dependent cascade initiated on endosome	1.4e-04	2.0e-07
MyD88-independent cascade	2.3e-06	1.2e-11
NFkB and MAP kinases activation mediated by TLR4 signalling repertoire	5.9e-05	6.0e-09
RIG-I/MDA5 mediated induction of IFN-alpha/beta pathways	2.6e-07	1.1e-07
Signaling by Interleukins	8.5e-13	6.8e-18
Toll Like Receptor 4 (TLR4) Cascade	1.2e-06	9.6e-12
Toll Receptor Cascades	2.6e-07	2.3e-13
TRIF mediated TLR3 signaling	6.0e-05	6.0e-09

Reactome pathway over-representation analysis of nodes within the CD8 or CD11b subnetworks. P-values are adjusted to control for multiple comparisons.

Next we examined the extent of node interconnection in the unbiased subnetworks in order to identify Hubs, which are defined as the most interconnected nodes within a network (nodes with the highest number of interactions). Hubs receive and integrate signals from multiple signal transduction pathways and are thus thought to be essential regulators or modulators of cell signalling. In the context of our in vivo LPS response model, the identified Hubs would be predicted to be integrating primary LPS-TLR4 and secondary immune signals to generate the observed immune outcomes. The degree of interconnection for each node was scored using the cytoscape plugin Cytohubba [43]. This analysis revealed that many Hubs (defined as nodes with more than 5 interactions) were the core LPS response molecules curated from the literature (Figure 5, Figure S2, Tables S5 and S6). Tlr4, Traf6, Ikbke, Irf7, Nfkbia, Fos, Jun, and Mapk1 were Hubs in both CD8 and CD11b DC subnetworks (Figure 5). When overlayed on the linear TLR4 KEGG pathway, these common Hubs mapped to both the Myd88-dependent and -independent pathways (Figure S5). This analysis further supports a model in which transcriptional responses to both primary and secondary signals are orchestrated via molecular pathways common to both subsets, consistent with the transcription factor and core signalling molecule expression data.

However, we did identify some core signalling molecules that were selectively present in only one of the 2 unbiased subnetworks. Thus the CD8 DC subnetwork uniquely contained Map3k7, Mapk11, and Tab2, while the CD11b DC subnetwork uniquely contained Ikbkg, Irak1, Mapk8, Myd88, Nfkb1, Rela, Ripk1, Tirap, Tollip, and Traf3 (Figure 5). Once again, these Hubs mapped to both the Myd88-dependent and -independent pathways (Figure S5), potentially suggesting that modulation of a ‘common’ signalling cascade occurs at different intervention points in the 2 subsets.

Given the large number of predefined core LPS response Hubs identified within both unbiased subnetworks, we analysed the subnetworks (Figure 5, Tables S5–S6) for the presence of the pathway modulators identified in the initial filtered analysis (Figure 4A, Table S4). A high degree of overlap was observed between the two analyses. 83% of the CD11b and 96% of the CD8 subset-regulated nodes identified in the filtered analysis were also identified in the unbiased subnetworks. In addition, many of these pathway modulators were among the most highly interconnected Hubs (defined as nodes with more than 15 interactions) within the unbiased subnetworks, or directly interacted with the most highly interconnected Hubs, consistent with a key functional role. Atf3, Ep300, Gadd45g, Ikzf4, Jak3, Relb, Tnfaip3 and Zbtb16, which were selectively present in the CD8 DC unbiased subnetwork, and Cdkn1a, Fth1, Gadd45a, Id2, Notch3, Serbinb9/Spi6, and Tradd in the CD11b DC subnetwork, are all known key modulators of cell signalling. Our analysis indicates for the first time that these molecules are also candidate modulators of DC subset-specific responses to LPS.

We also identified a number of additional cell surface receptors including IL-7R and CXCR4 as Hubs (5–20 interactions) present within the subnetworks of both subsets (Figure 5). These receptors are known to recognise the LPS-inducible ligands IL-7 and CXCL12, respectively [44]–[47], and their presence within both signalling subnetworks is consistent with an important role for secondary immune mediators in influencing the observed transcriptional responses in the context of the immune microenvironment.

**Figure 6:**
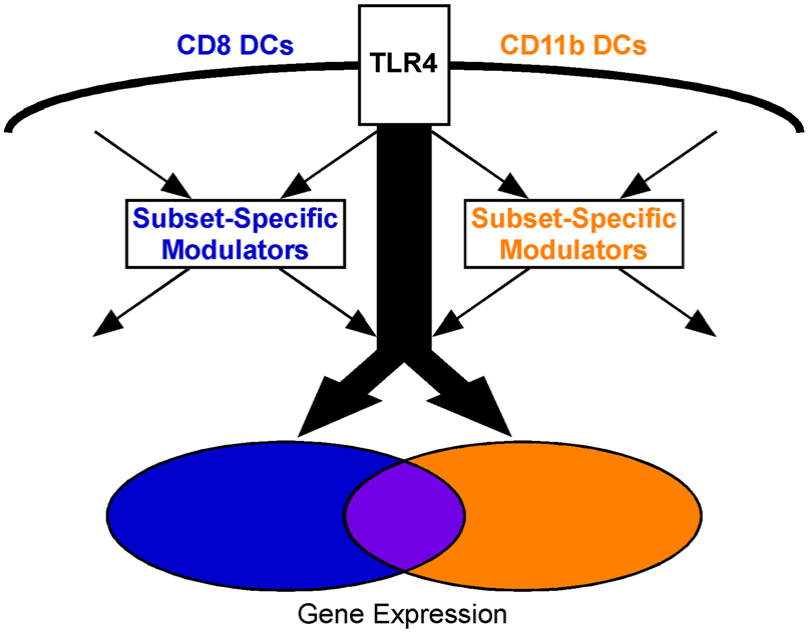
The pathway modulation model. Schematic representation of our proposed model. LPS-induced signaling occurs through a common set of signaling molecules. LPS induces the expression of subset-specific TLR4 pathway modulators, which ‘fine tune’ signaling and allow for distinct immune outcomes in closely related cell subsets. These pathway modulators likely integrate both signals derived directly from TLR4 and exogenous signals from the microenviroment that contribute to the subset-specificity of the response.

Based on these results, we suggest that LPS responses are regulated through a set of common pathway molecules in both DC subsets. Subset-specific responses are achieved by differential regulation of known pathway modulators that subsequently ‘fine-tune’ signalling by means of their interactions with common Hubs and multiple other signalling molecules involved in primary (LPS-TLR4) and secondary cytokine/chemokine/cell-cell interaction pathways (Figure 6).

### The model of subset-specific LPS response regulation is applicable to non-DC cell populations

To test whether regulation of transcriptional responses to LPS in other cell subsets is consistent with our model, we reanalysed published datasets for which two distinct but related cell subsets were stimulated with LPS in the same experiment (datasets 16–19 listed in Table S1). This meta-analysis included paired datasets comparing mouse thioglycate-elicited peritoneal macrophages with bone marrow-derived macrophages (Figure 4B, Table S7, [26]), human Vδ1 versus Vδ2 γδ T cells (Figure 4C, Table S8, [40]), human retinal vascular versus choroidal endothelial cells (Figure 4D, Table S9, [41]), and cord-blood monocytes compared with neutrophils (Figure 4E, Table S10, [48]) These datasets all used in vitro stimulated cells and thus measured only primary TLR4-mediated outcomes.

We repeated the filtered and unbiased network analyses, as described above, on datasets 16–19. Consistent with our DC data (Figure 4A), subset-specific gene regulation was seen in every paired dataset (Figure 4B–E, Tables S7–S10). Once again, there was a large overlap between the filtered and unbiased analyses, and many of the most interconnected Hubs in each of the unbiased subnetworks were either core TLR4 signalling molecules or candidate modulators identified in the original filtered analysis (Tables S11–S18). As predicted by our model, many of these modulators were selectively present in only one of two paired subnetworks (Tables S11–S18).

## Discussion

Dendritic cells are essential in triggering and tailoring the adaptive immune response. Understanding how defined DC subsets differentially respond to pathogenic signals is a crucial step in understanding how subset-specific control of immune outcomes is achieved. To address this question, we employed systems biology approaches to globally characterise the responses of splenic CD8 and CD11b DC subsets following LPS stimulation. Our methodology focused on identifying sets of modulators (the product of network analysis) rather than specific genes, and was specifically designed for the analysis of single pooled samples. As our conclusions are based on a self-reinforcing network analysis [3]–[5], combined with a hyper-stringent method for choosing differentially expressed genes [33], the need for multiple replicates was reduced compared to methodologies considering individual gene expression events. In addition, we used extensive meta-analyses to validate the quality of our data in the absence of technical replication (Figures 1C, 3, Table S1).

Using RNA-Seq on cells isolated ex vivo from pooled biological replicate animals and immediately FACS sorted, we have shown that CD8 and CD11b DC subsets respond uniquely following in vivo LPS stimulation (Figure 2). We have defined new subset-specific biomarkers and mechanistic information in addition to confirming previously identified differences in surface marker expression and cytokine secretion following LPS stimulation in vitro and in vivo [34], [37], [49]. One unique finding from our analysis was the unexpectedly wide range of cytokines/chemokines and co-stimulatory molecules differentially regulated in CD11b and CD8 DCs (Figure 2C). This might indicate an inherent difference in the use of soluble versus cell-to-cell dependent mechanisms for modulating immune responses in the 2 DC subsets. Our analysis also implicates differential responsiveness to external stimuli as one of the mechanisms by which DC subsets regulate their functionality. To our knowledge, this is the first comprehensive examination of the inflammatory transcriptional response in well-defined DC subsets in vivo. In contrast to a recent report in which steady-state plasmacytoid DCs, conventional DCs, and the CD8 DC subset were individually subjected to network analysis of only those genes uniquely expressed by DCs, we included all expressed genes in a direct comparison of subsets, thus highlighting the behaviour of key functional molecules expressed in common with a range of other cell populations [20]. Several other groups have also identified core modulatory molecules within immune response pathways, but have focused on related cell types, rather than subsets within a single cell type [50]–[54].

A major implication of our study is that the transcriptional response to LPS is DC subset specific, and that analysis of unfractionated populations will by definition over-generalise such responses. To highlight this concept, we compared LPS responsive genes in our study to those identified in published datasets. As predicted, datasets from in vitro stimulated BM-DCs and in vivo stimulated unfractionated CD11c^+^ DCs included many LPS responsive genes that were regulated in a subset-specific manner within our study (Figure 3A–C). This observation has major relevance to our understanding of responses to LPS, which has usually been defined by the study of mixed populations, and has often been generalised/extended across additional populations without experimental verification. Our study suggests that transcriptional control of signalling networks may need to be refined in a subset-specific context.

While we observed a high degree of overlap with published datasets from LPS-stimulated BM-DCs, over 30% (221 out of 705) of the LPS responsive genes identified in our study had not previously been identified in any BM-DC dataset (Figure 3A), while 73% were not identified in the BM-DC dataset from the same timepoint (24hr) as our study (Figure 3B). This may be due to many factors, including the influence of additional signals in the splenic microenvironment, differences in the starting DC population, experimental variables such as the use of microarrays and/or differences in experimental set-up/reagents, or a combination of these factors. GM-CSF-induced BM-DCs are known to comprise a mixed population of ‘inflammatory’ DCs (CD24- and CD11b-expressing subsets) that poorly reflect the in vivo steady-state DC populations in terms of surface marker expression and cytokine secretion [36]–[38]. Thus the comparison between BM-DCs and steady-state splenic DC subsets is complicated both by the poor correlation between populations and by the ‘mixed’ nature of the BM-DC population. Despite this, our results suggest the use of murine BM-DCs as model ‘DCs’ is failing to capture the subset-specificity of LPS responses within the DC compartment. Fms-like tyrosine kinase 3 ligand (Flt3L) may support a more physiological in vitro model for investigating DC signalling in a subset-specific manner, since Flt3L-generated BM-DCs more closely resemble the splenic CD8 DC [36]. Similar to the BM-DC comparisons, the disparity between our findings and the published analysis of in vivo LPS-stimulated CD11c^+^ DCs (Figure 3C) may in part be explained by experimental differences including the addition of anti-CD40 to the LPS stimulus, combined with the much shorter stimulus time (6 hr versus 24 hr) [15].

To explore how subset-specific differential responses to LPS stimulation may arise, we performed network analysis on LPS-regulated genes in the 2 DC subsets. We initially defined a set of 48 core LPS response molecules based on their well established roles in propagating and modulating LPS responses. The lack of gross signalling differences between the subsets was indicated by comparable expression of the vast majority of the 48 core molecules in the 2 subsets (Figure S2). Unbiased subnetwork analysis revealed that many of the core LPS response molecules were also present as core Hubs within the unbiased subnetworks (Figure 5), while Reactome pathway analysis indicated that both the Myd88-dependent and -independent pathways were highly enriched within the both subnetworks (Table 2). Thus it appeared that primary LPS-dependent signalling was likely to be similar in the 2 DC subsets. One notable exception was the increased steady-state expression of the signalling adaptor Ticam2 (Trif) in CD11b compared with CD8 DCs (Figure S2), which might suggest a bias in CD11b DCs towards signalling via the ‘Myd88-independent pathway’ mediated by Trif. However overlaying the core LPS signalling molecules within the CD11b subnetwork (Ikbkg, Irak1, Mapk8, Myd88, Nfkb1, Rela, Ripk1, Tirap, Traf3, and Tollip) onto the KEGG TLR4 pathway (Figure S5) indicated a distribution across both Myd88-dependent and -independent arms. This was also the case for the subset of core molecules common to both networks (Ikbke, Irf7, Fos, Jun, Mapk1, Nfkbia, and Traf6), once again supporting a model in which both subsets share the same primary TLR4-dependent signalling machinery. The three core molecules uniquely present in the CD8 subnetwork (Map3k7, Mapk11, and Tab2) mapped to the Myd88-dependent pathway. However CD8 DCs have previously been shown to signal through Trif after polyI:C stimulation, indicating that they also possess a functional Myd88-independent pathway [55].

The transcription factor-dependence of the differentially expressed genes was also comparable between the 2 DC subsets (Table 2). The identified transcription factors, including Nfkb1, Rela and Jun (AP-1), are all known to adopt key roles in functional aspects of the LPS response [6]–[9]. Although Irf8 was expressed at consistently higher levels in CD8 DCs (Table 2, Figure 1B) and is known to be critical for CD8 but not CD11b DC development [56], genes regulated by Irf8 were highly enriched in both CD11b and CD8 subsets, consistent with the known impairment of responses to CpG and LPS in Irf8-deficient CD11b DCs [56]. Collectively, these data suggests that both DC subsets utilise a common core pathway, characterised by a well established set of signalling Hubs and transcription factors.

The absence of clear differences in known pathway components supports a more subtle mechanism of signal modulation (Figure 6). Both filtered and unbiased network analyses clearly identified sets of genes with known regulatory or modulatory function uniquely present within the subset-specific subnetworks (Figures 4A, 5, Tables S4–S4). We hypothesize that LPS-dependent transcriptional regulation of these genes mediates the observed subset-specific differences in the response to the combination of primary (LPS-TLR4) and secondary immune signals to which the DC subsets are exposed in vivo. Within CD8 DCs, the GO term ‘negative regulation of the inflammatory response’ was over-represented, supported by the identification of known negative regulators Atf3, Tnfaip3 (A20), and Zbtb16 (PLZF) as key hubs in the network analysis, and suggesting that negative regulation may be a hallmark of the CD8 subset [57]–[59]. Interestingly, Atf3 is a predominant negative regulator of inflammation [51] and has been previously identified as one of the most important molecules identified within the signalling network of LPS-stimulated bone-marrow macrophages [54]. Differential regulation of MAPK signalling might by another mechanism by which DC subsets mediate subset-specific responses, as Gadd45g (uniquely identified in the CD8 subnetwork) and Gadd45a (uniquely identified in the CD11b subnetwork) are known to differentially modulate MAPK pathways in hepatoma cells [60]. Similarly, the CD11b subnetwork uniquely contained TRADD, which is known to play a differential role in regulating NFκB and MAPK signalling in fibroblasts versus macrophages and is a likely key mediator of the unique CD11b DC LPS-response [61]. Cdkn1a (p21/WAF1/CIP1), also exclusively present within the CD11b subnetwork, was another highly interconnected Hub that is known to be essential for regulating LPS activation in macrophages [62], [63]. Thus our analysis has revealed previously unappreciated subset-specific roles for several known signalling modulators.

We propose that our model in which the fine-tuning of central immune pathways mediates subset specific responses is indeed relevant in a physiological context since our data were obtained from cells taken directly from mice with minimal manipulation.

Our experimental design characterising ‘late’ in situ DC subset responses to LPS provides a more physiological model than an ex vivo stimulation assays. However, while both DC subsets can respond directly to LPS [31], our analysis is complicated by the integrated response of the DC subsets to additional exogenous signals arising from the splenic microenviroment. In agreement with this, we identified differential expression of a number of additional cell surface receptors within the unbiased subnetworks of the 2 DC subsets (Figure 5). This suggests that both DC subsets are integrating direct (LPS-TLR4) signals with those from other secondary immune mediators such as IL7 (via IL7R) and CXCL12 (via CXCR4), which are known to play multifunctional roles in regulating DC function [44]–[47]. However, the greatest strength of unbiased subnetwork analysis is the ability to identify key functional molecules (Hubs) within complex signalling networks, without relying on previously defined linear pathways. Therefore, the core LPS response molecules and pathway modulators identified here as Hubs are likely responsible for integrating signals derived directly from TLR4 with additional exogenous signals, resulting in the observed subset-specific responses. Indeed a major strength of this study is the physiological, complex nature of the in vivo stimulus, and its potential to serve as a basis for identifying key secondary immune signals regulating DC function following LPS exposure (such as CXCL12-CXCR4). However, further studies are needed to fully characterise how the observed subset-specific responses are controlled in the context of the immune microenvironment.

While we have identified many candidate genes potentially involved in regulating subset-specific responses (as discussed above), our hypothesised model is primarily based on sets of subset-specific modulators regulating common signalling pathways, rather than these individual genes. This ensures that our conclusions are relatively resistant to errors introduced by using a single pooled sample for each condition. As with any transcriptional-based study, complex functional studies, such as those using knockout/knockin models, will be required to validate individual genes that are actively regulating subset-specific function. However, we believe that our approach represents an important first step in understanding how cell subsets may regulate their responses to common stimuli.

Given our focus on sets of modulators, we chose to validate the suitability of our hypothesised model of pathway modulation and signal integration, rather than the expression of individual genes. To do this, we tested how well our model fitted to published datasets for both mouse and human cell subset responses to LPS. In each dataset, we identified cell subset-specific modulators that are known to interact with core LPS response molecules and which uniquely act as Hubs within their respective signalling networks (Tables S7–S10). While we cannot fully exclude other mechanisms contributing to the observed subset differences in these published datasets, the results strongly support our current model. Thus pathway modulation appears to represent a global mechanism allowing for tightly controlled and specific responses to LPS in related but distinct cell populations.

While we have subdivided DCs in this study on the basis of CD8 and CD11b expression, multiple reports have suggested that the splenic DC network is much more complex and that splenic DCs can be further subdivided based on the expression of many additional surface markers including ESAM1 [64], DCAL2 [65], CD207/Langerin [66], CD103 [67] and/or CD205/DEC-205 [68]. Our model would predict that further subdivision on the basis of these markers would reveal additional layers of complexity in the subset-specific regulation of LPS responses. Further complexity also arises from the dynamic nature of LPS responses over time [13], [39], [53]. Previous network analysis of time course responses has shown that the majority of Hubs are only transiently involved as key regulators under certain conditions, and that even permanent Hubs redefine their interactions dynamically [5]. Since we consistently identified differential subset-specific regulation of TLR4-interacting proteins in datasets from multiple timepoints and cell populations in mouse and human (Figure 4, Tables S7–S10), it is likely that this means of signalling modulation and fine tuning plays a critical role throughout the response.

## Materials and Methods

### Mice and treatment

All mice were housed under specific pathogen-free conditions in the Centenary Institute Animal Facility. [C57BL/6× B10.BR]F1 mice on a CD45.1/CD45.2 heterozygous background were used for all experiments. Our unpublished results have identified no differences in phenotype and function of splenic DC subsets isolated from [C57BL/6× B10.BR]F1 mice and their C57BL/6 counterparts. Mice were injected intraperitoneally with 25 μg LPS (rough strains from *Salmonella enterica* serotype Minnesota Re 595, Sigma-Aldrich) per mouse. Animals were sacrificed mice 24 hours after LPS injection.

### Ethics Statement

Approval for all animal experimentation was obtained from the Animal Ethics Committee at the University of Sydney.

### Flow cytometry and cell sorting

Spleens were pooled from 5 (control) or 10 (LPS-stimulated) animals, digested with 2 mg/ml Collagenase IV from *Clostridium histolyticum* (Sigma-Aldrich) and a single cell suspension prepared as described previously [69]. Cells for RNA-Seq were prepared by cell sorting. Briefly, splenocytes were stained for B220 (clone RA3-6B2) prior to DC selection. DCs were isolated with anti-CD11c MicroBeads (clone N418, Miltenyi Biotec) after enrichment for Lineage (Ter119/B220/CD19/CD3/Gr-1)-negative cells using rat anti-mouse antibodies and anti-rat IgG MicroBeads (Miltenyi Biotec). Selected DCs (78% purity) were stained for CD11c (clone HL3), CD11b (clone M1/70), CD8 (clone 53–6.7) (all from BD/Pharmingen) and pan-MHCII (clone M5/114, eBioscience). Staining was performed in PBS containing 5% FCS and 10mM EDTA, non-specific staining was blocked with unconjugated anti-CD16.32 (clone 2.4G2) and DAPI was used to exclude dead cells. B220^−^MHCII^+^CD11c^+^ cells were sorted into CD11b^+^ and CD8^+^ subsets on Aria-Ilu (BD) to 99.1% and 86.3% purity, respectively). For analysis, DAPI-negative (live cells) events were gated on forward scatter height vs. area to exclude doublets. CD86 expression was detected using anti-CD86 (clone GL1, BD/Pharmingen). To identify monocyte-derived DCs, cells were additionally stained with anti-FcεR1α (clone MAR-1, eBioscience), CD64/FcγR1 (clone X54-5/7.1, BD/Pharmingen) and Gr-1 (unconjugated, detected with anti-Rat-Alexa488, Invitrogen).

### RNA isolation and RNA-Seq

Total RNA was isolated from sorted DCs (>98% purity in every sample) using RNeasy Micro Kits (Qiagen). A minimum of 200,000 cells and 100 ng of RNA per condition were used for RNA-Seq. RNA quality was determined using a Bioanalyzer 2100 (Agilent Technologies), and cDNA libraries were prepared from total RNA according to the Illumina TruSeq RNA sample preparation guide. Unique adapter indexes (Illumina) were attached during sample prep and samples were run pooled and loaded into a single flow cell lane to reduce technical variability. RNA-Seq was performed on a GAIIx instrument (Illumina), using a single read run with 36 amplification cycles (29 sample +7 adapter/index sequence).

### Data Processing

Raw basecall data was converted to FASTQ sequence files using Off-Line Basecaller (Ilumina) and a custom Perl script. Reads were aligned to the mm9 mouse genome with TopHat version 2.05 and Bowtie1 version 0.12.7 [70]. Reads were initially mapped to Ensembl transcripts with the search for novel junctions disabled, using standard Tophat filtering/stringency parameters [70]. Genomic coordinates were then transformed into counts of protein-coding Ensembl genes. To do this, a chimeric gene-model was first defined by merging all protein-coding transcripts for a given gene. Transcripts that had reads in less than 50% of their exons in all samples were defined as not expressed and were excluded from the chimeric transcriptome. Reads that overlapped the chimeric genes were counted using the htseq-count script in the intersection-nonempty mode (http://www-huber.embl.de/users/anders/HTSeq/doc/count.html). The script discards multi-mapped reads as well as reads that overlap multiple distinct genes, to generate a file of uniquely mapped gene counts. No additional gene filtering was performed. Total mapped reads to protein coding genes were 6702672 for steady state and 5536485 for LPS-stimulated CD8 DCs, and 6368840 for steady state and 2582238 for LPS-stimulated CD11b DCs.

### Transcriptional Analysis

Analysis of RNA-Seq gene count data was performed in R using Bioconductor ([71] r02290). Differential gene expression was calculated using the DESeq package [33]. DESeq was run with the method = blind and sharingMode = fit-only settings for single-replicate experiments. This sets all samples as replicates of each other when calculating the variance. This method tends to be over-conservative as compared to a replicated experiment ([33] r02353). Genes with an associated p-value ≤0.05 were scored as differentially expressed between samples. A full list of expressed genes is provided in Table S19. Gene ontology (GO) and transcription factor-target over-representation analyses were calculated using the Wallenius distribution in the goseq package, which normalises for RNA-Seq length biases ([72] r02354). Tests in which fewer than 10 genes in a term were observed in both subsets were excluded from further analysis. The threshold of significance in the ORA tests was defined as a Benjamini-Hochberg [73] adjusted p-value ≤0.05. Odds-Ratios were calculated as the odds of a differentially expressed gene occurring in the ORA category divided by the odds of a non-differentially expressed gene occurring in the ORA category, so that numbers greater than 1 are considered to reflect associations that are likely to be real. GO-Terms to gene mappings and Reactome Pathway to gene mappings were obtained using the biomaRt package [74]. Transcription factor to gene mappings were downloaded from InnateDB after searching for protein-gene interactions between all genes (including those predicted by orthology) [42]. RNA-Seq data was deposited in the NCBI Gene Expression Omnibus (GEO) repository (GSE42573).

### Network analysis

Differentially expressed (DE) genes from multiple analyses were uploaded separately into InnateDB [42], a specialised interactome database containing all known protein-protein interactions but highly curated for immune protein reactions, to generate a list of interactions between DE genes in the dataset, and with first-order non-DE proteins with curated experimental evidence of an interaction. Interactions predicted by orthology were included in all analyses. To analyse the complex functional relationships between genes comprising the observed LPS stimulation signatures, regulated genes were viewed as nodes in a network (“network analysis”), connected to one-another by their protein-level interactions (edges), as previously described [75], [76]. Briefly, interaction networks were visualised in Cytoscape [77] using the Cerebral plugin to show gene location relative to the cell [78]. These networks were filtered for protein-protein interactions after removing duplicate edges, self-loops and the general ubiquitin, Ubc. Ubc interacts with ∼3000 proteins and its inclusion thus biases subsequent subnetwork analyses. Subnetwork analysis was carried out on each of the networks using the jActive plugin for Cytoscape, and the top significant subnetworks were ranked on the basis of their calculated Z-score [79], [80]. Multiple significant subnetworks were merged for some comparisons based on their high degree of overlap and associated z scores within a stratum. Highly interconnected gene nodes within these subnetworks are referred to as Hubs, and represent key molecules involved in signal trafficking. While the term “Hub” cannot be rigorously defined in the context of computational network analysis, we have defined Hubs as nodes with 5 or more interactions and the “most highly interconnected” Hubs as nodes with 15 or more interactions. Hub degree was scored using the Cytoscape software plugin cytoHubba with the higher scoring nodes predicted to represent essential molecules [43].

In separate filtered analyses, core signalling molecules in the TLR4 pathway in mouse and human were uploaded into InnateDB to generate a list of 2279 first-order interacting proteins [42]. This network was filtered for interacting nodes significantly differentially expressed in a cell subset and visualised as a venn diagram representing each interacting nodes' differential expression in one or both of the subsets.

### Meta-Analyses

Normalised datasets were downloaded from NCBI GEO using the Bioconductor package GEOquery [81]. GSE15907 and GSE32381 were downloaded manually from NCBI GEO as raw CEL files, quantile normalised and RMA background corrected. Datasets were included in our analyses if they contained at least two biological replicates (see Table S1 for a list of reanalysed datsets). Differential expression was calculated using the limma package. Genes were defined as significant at a Benjamini-Hochberg [73] adjusted p-value <0.05. A minimum fold change cut-off of 2 was applied for network analysis. Genes from γδ T cells (GSE3720), endothelial cells (GSE7850), and cord blood monocytes and neutrophils (GSE39840) were defined significant at a non-adjusted p-value cut-off of 0.05 and a fold-change cutoff of 2. To compare our RNA-Seq study to previously published microarray datasets characterising steady-state splenic DC subsets (datasets 1–9 listed in Table S1), we used a hypergeometric test approach. To do this, we first identified those genes in each dataset that were significantly differentially expressed in CD8 versus CD11b DCs at a p-value cut-off of 0.05. We then used a hypergeometric test to calculate the significance of overlap between each of these gene lists and the gene list derived from our RNA-Seq study.

## Supporting Information

Figure S1 **DC purification strategy excludes monocytes and inflammatory monocyte-derived DCs**. Spleen cells from control and LPS-injected mice were subjected to the pre-sort bead selection procedure as described in Materials and Methods. Selected cells were stained for MHCII, CD11c, CD64/FcγR1, FcεR1α, Gr-1 and CD11b, and analysed for the presence of contaminating monocytes, inflammatory monocytes and monocye-derived DCs. (A) The lineage (CD19, B220, CD3, Gr-1, Ter119)-negative MHCII^+^CD11c^+^ gating strategy excludes FcγR1^+^FcεR1α^+^ monocyte-derived DCs. (B) Conversely, monocytes and inflammatory monocytes expressing Gr-1 do not significantly contaminate the MHCII^+^CD11c^+^ sorting gate shown in the right panels.
(TIFF)

Figure S2 **Expression of core LPS response molecules.** Comparison of core LPS response molecules in CD8 and CD11b DCs in the steady-state (−LPS) and after LPS stimulation (+LPS). Data are presented as fold changes (CD11b/CD8). * Significantly differentially expressed before LPS stimulation; ** Significantly differentially expressed after LPS stimulation.
(TIFF)

Figure S3 **Network analysis of LPS-responsive genes in CD8 DCs.** A network analysis was carried out on the transcriptional response of CD8 DCs stimulated in vivo with LPS as compared to steady-state. Subnetwork analysis was used to enrich networks in an unbiased manner for interactions with differentially expressed genes. The figure was made using the Cytoscape plugin Cerebral to show the cellular localisation of each gene. The size of each node is proportional to its Hub degree (interconnectivity with other genes), while node colour indicates relative gene expression (+LPS/−LPS). Square nodes represent core LPS response molecules. Nodes labelled in blue text are present in the CD8 but not CD11b DC subnetwork, while nodes labelled in black text are present in both. Networks were organised using the Cytoscape plugin Cerebral, which organises nodes based on their relative cellular location. For visualisation, only selected nodes are labelled. The full list of nodes/network characteristics is provided in Table S5.
(TIFF)

Figure S4 **Network analysis of LPS-responsive genes in CD11b DCs.** A network analysis was carried out on the transcriptional response of CD11b DCs stimulated in vivo with LPS as compared to steady-state. Subnetwork analysis was used to enrich networks in an unbiased manner for interactions with differentially expressed genes. The figure was made using the Cytoscape plugin Cerebral to show the cellular localisation of each gene. Node size is proportional to its Hub degree (interconnectivity with other genes/nodes), and node colour indicates relative gene expression (+LPS/−LPS). Square nodes represent core LPS response molecules. Nodes labelled in orange text are present in the CD11b but not CD8 DC subnetwork, while nodes labelled in black text are present in both. Networks were organised using the Cytoscape plugin Cerebral, which organises nodes based on their relative cellular location. For visualisation, only selected nodes are labelled. The full list of nodes/network characteristics is provided in Table S6.
(TIFF)

Figure S5 **Subset-specific Hubs in relation to a KEGG pathway map of TLR signalling.** Core LPS response Hubs identified in the subnetwork analysis of CD8 or CD11b are identified by coloured dots and gene names (italics) overlayed on a KEGG pathway map. Black dots and text indicate Hubs identified in both subnetworks, blue indicates Hubs identified only in the CD8 subnetwork and orange indicates Hubs identified only in the CD11b subnetwork.
(TIFF)

Table S1 **List of reanalysed datasets and their associated references.**
(DOCX)

Table S2 **GO term over-representation analysis on nodes within the CD8 or CD11b subnetworks.** P-values are adjusted to control for multiple comparisons.
(CSV)

Table S3 **Differentially-expressed genes identified by comparing LPS stimulated with steady state expression data for each DC subset.**
(XLSX)

Table S4 **Gene list of differential pathway modulators in CD8 and CD11b DCs from this RNA-Seq study, as depicted in** **Figure 4A** **.**
(XLSX)

Table S5 **Full node lists and corresponding network characteristics for the subnetwork of LPS-responsive genes in CD8 DCs.**
(XLSX)

Table S6 **Full node lists and corresponding network characteristics for the subnetwork of LPS-responsive genes in CD11b DCs.**
(XLSX)

Table S7 **Gene list of differential pathway modulators in thioglycolate-elicited peritoneal macrophages and bone-marrow derived macrophages, as depicted in** **Figure 4B** **.**
(XLSX)

Table S8 **Gene list of differential pathway modulators in Vδ1 and Vδ2 γδ T cells, as depicted in** **Figure 4C** **.**
(XLSX)

Table S9 **Gene list of differential pathway modulators in retinal vascular endothelium and choroidal endothelial cells, as depicted in** **Figure 4D** **.**
(XLSX)

Table S10 **Gene list of differential pathway modulators in cord blood monocytes and neutrophils, as depicted in** **Figure 4E** **.**
(XLSX)

Table S11 **Full node lists and corresponding network characteristics for the subnetwork of LPS-responsive genes in thioglycolate-elicited macrophages.**
(XLSX)

Table S12 **Full node lists and corresponding network characteristics for the subnetwork of LPS-responsive genes in bone-marrow derived macrophages.**
(XLSX)

Table S13 **Full node lists and corresponding network characteristics for the subnetwork of LPS-responsive genes in Vδ1 γδ T cells.**
(XLSX)

Table S14 **Full node lists and corresponding network characteristics for the subnetwork of LPS-responsive genes in Vδ2 γδ T cells.**
(XLSX)

Table S15 **Full node lists and corresponding network characteristics for the subnetwork of LPS-responsive genes in retinal vascular endothelial cells.**
(XLSX)

Table S16 **Full node lists and corresponding network characteristics for the subnetwork of LPS-responsive genes in choroidal endothelial cells.**
(XLSX)

Table S17 **Full node lists and corresponding network characteristics for the subnetwork of LPS-responsive genes in cord blood monocytes.**
(XLSX)

Table S18 **Full node lists and corresponding network characteristics for the subnetwork of LPS-responsive genes in cord blood neutrophils.**
(XLSX)

Table S19 **All differential expression data from this RNA-Seq study.**
(CSV)
