# Supplementary figures and images for: A Systems Biology Approach to the Analysis of Subset-Specific Responses to Lipopolysaccharide in Dendritic Cells
Source: PLoS One. 2014 Jun 20;9(6):e100613. doi: 10.1371/journal.pone.0100613 (PMC4065045; doi:10.1371/journal.pone.0100613)

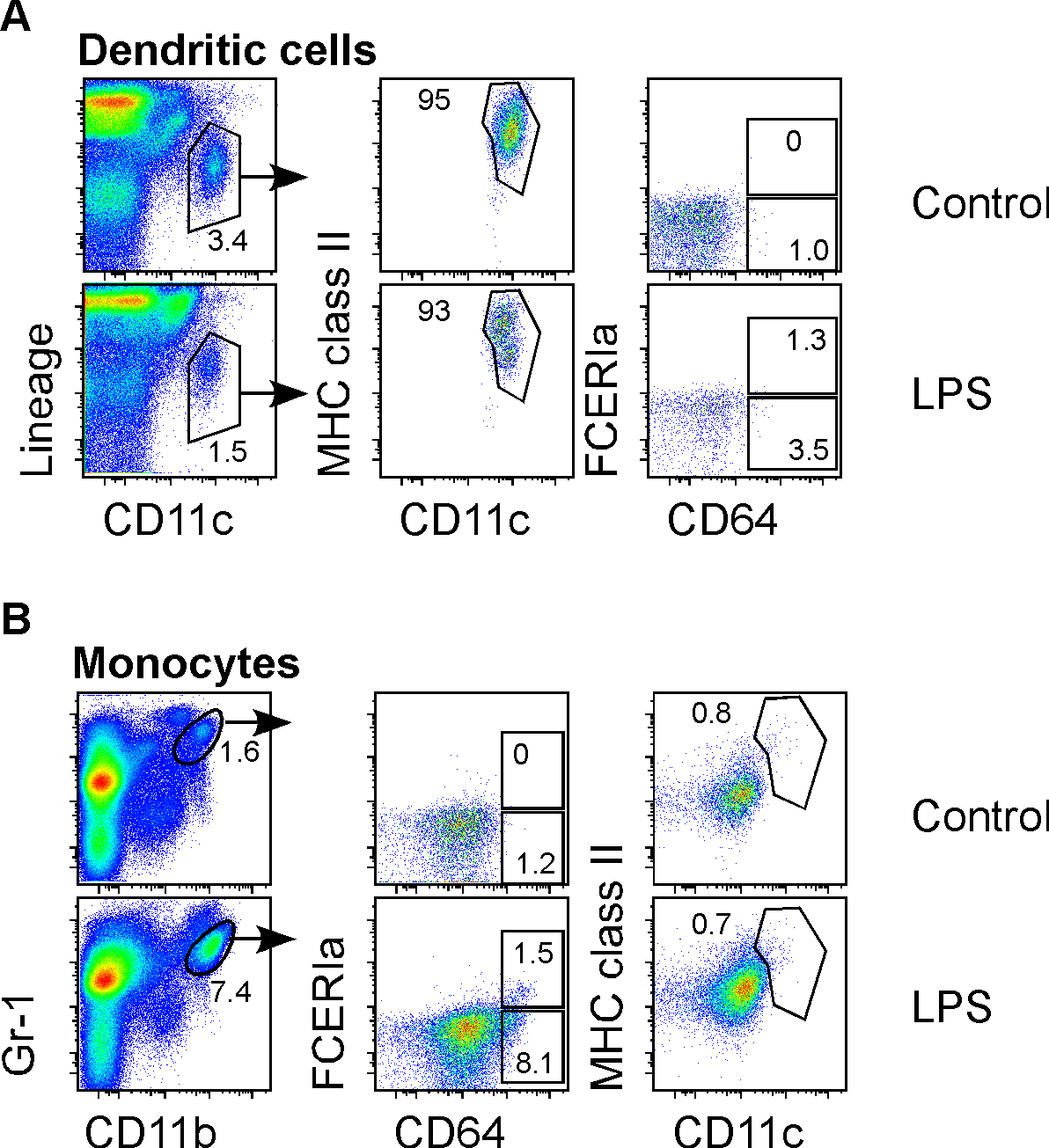

Supplement: Figure S1 — DC purification strategy excludes monocytes and inflammatory monocyte-derived DCs. Spleen cells from control and LPS-injected mice were subjected to the pre-sort bead selection procedure as described in Materials and Methods. Selected cells were stained for MHCII, CD11c, CD64/FcγR1, FcεR1α, Gr-1 and CD11b, and analysed for the presence of contaminating monocytes, inflammatory monocytes and monocye-derived DCs. (A) The lineage (CD19, B220, CD3, Gr-1, Ter119)-negative MHCII+CD11c+ gating strategy excludes FcγR1+FcεR1α+ monocyte-derived DCs. (B) Conversely, monocytes and inflammatory monocytes expressing Gr-1 do not significantly contaminate the MHCII+CD11c+ sorting gate shown in the right panels. (TIFF) [file pone.0100613.s001.tif]

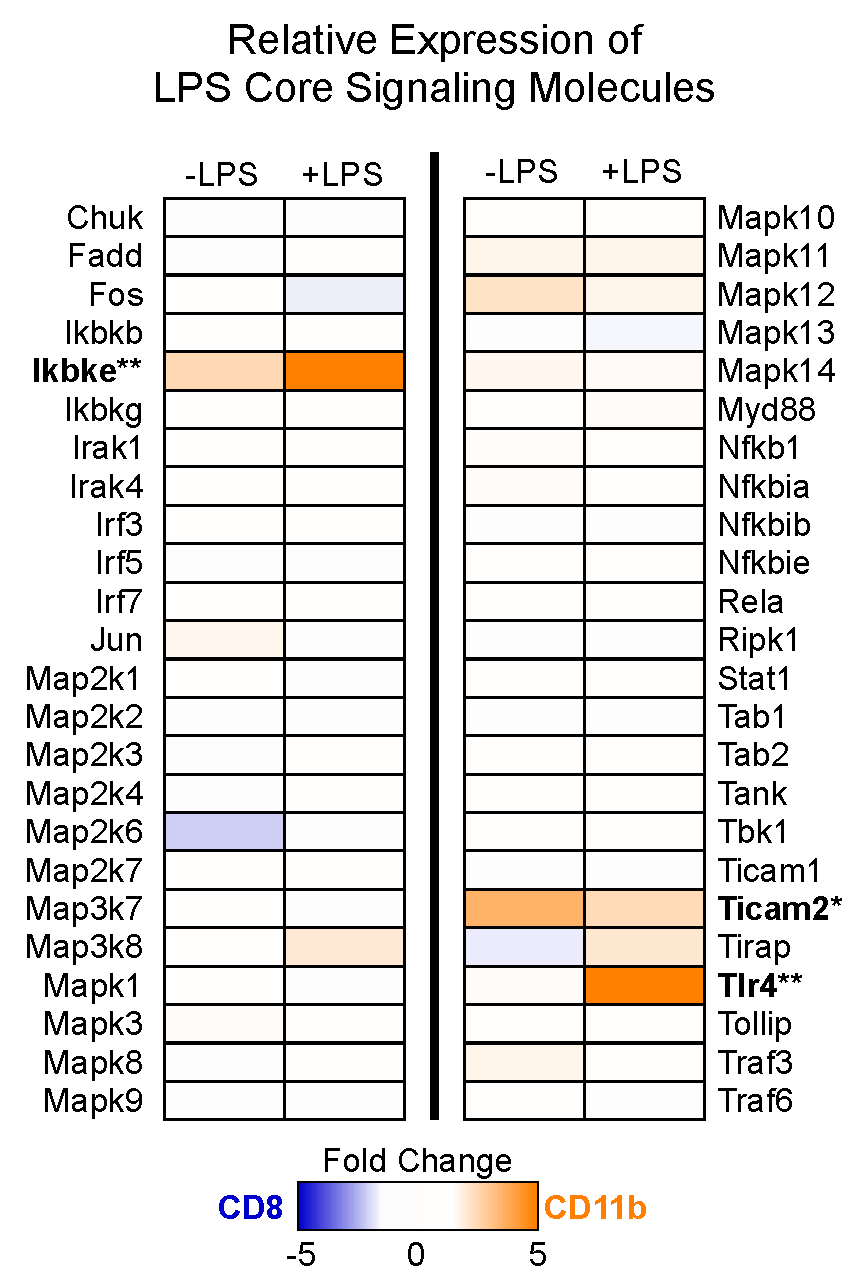

Supplement: Figure S2 — Expression of core LPS response molecules. Comparison of core LPS response molecules in CD8 and CD11b DCs in the steady-state (−LPS) and after LPS stimulation (+LPS). Data are presented as fold changes (CD11b/CD8). * Significantly differentially expressed before LPS stimulation; ** Significantly differentially expressed after LPS stimulation. (TIFF) [file pone.0100613.s002.tif]

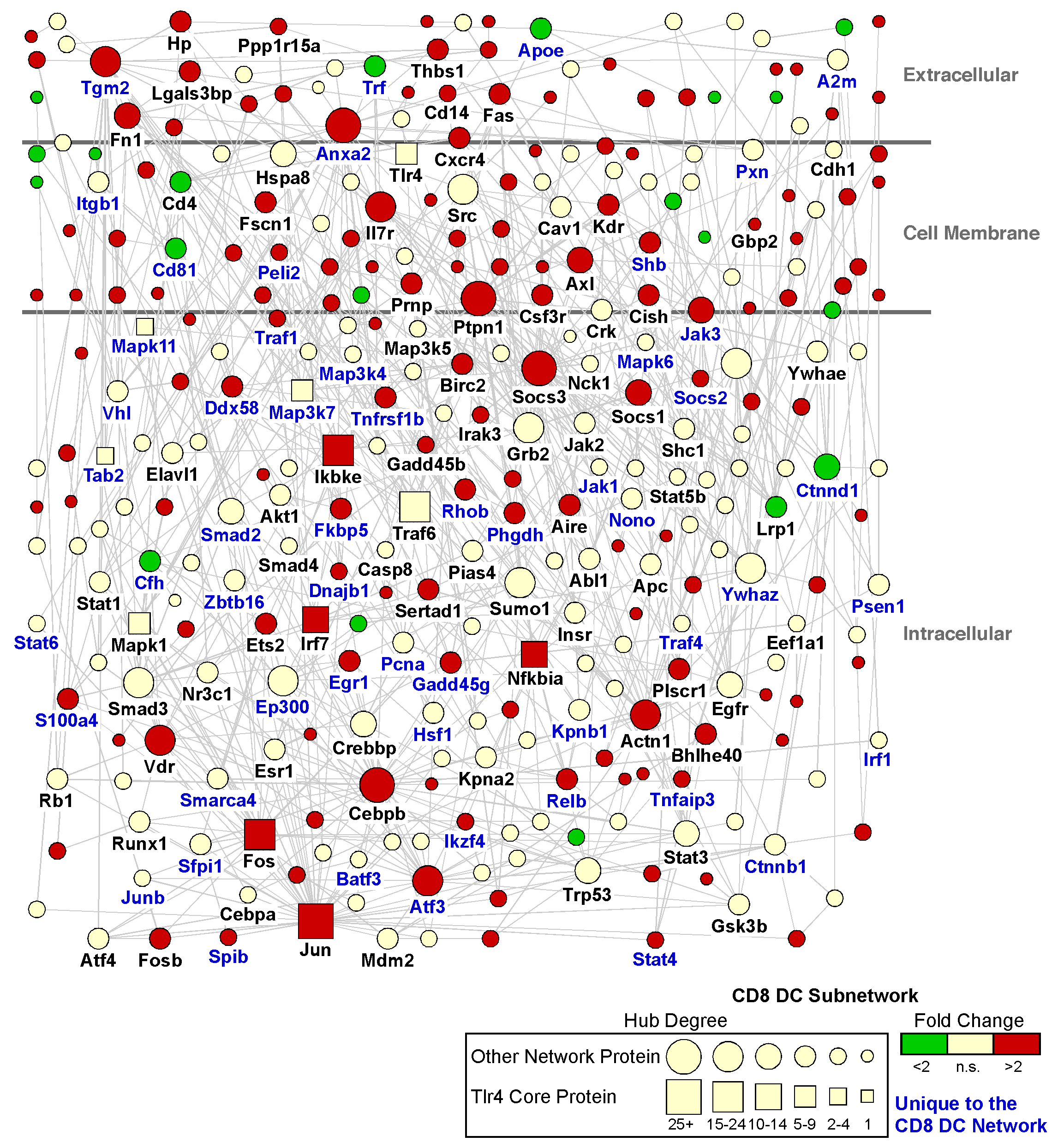

Supplement: Figure S3 — Network analysis of LPS-responsive genes in CD8 DCs. A network analysis was carried out on the transcriptional response of CD8 DCs stimulated in vivo with LPS as compared to steady-state. Subnetwork analysis was used to enrich networks in an unbiased manner for interactions with differentially expressed genes. The figure was made using the Cytoscape plugin Cerebral to show the cellular localisation of each gene. The size of each node is proportional to its Hub degree (interconnectivity with other genes), while node colour indicates relative gene expression (+LPS/−LPS). Square nodes represent core LPS response molecules. Nodes labelled in blue text are present in the CD8 but not CD11b DC subnetwork, while nodes labelled in black text are present in both. Networks were organised using the Cytoscape plugin Cerebral, which organises nodes based on their relative cellular location. For visualisation, only selected nodes are labelled. The full list of nodes/network characteristics is provided in Table S5. (TIFF) [file pone.0100613.s003.tif]

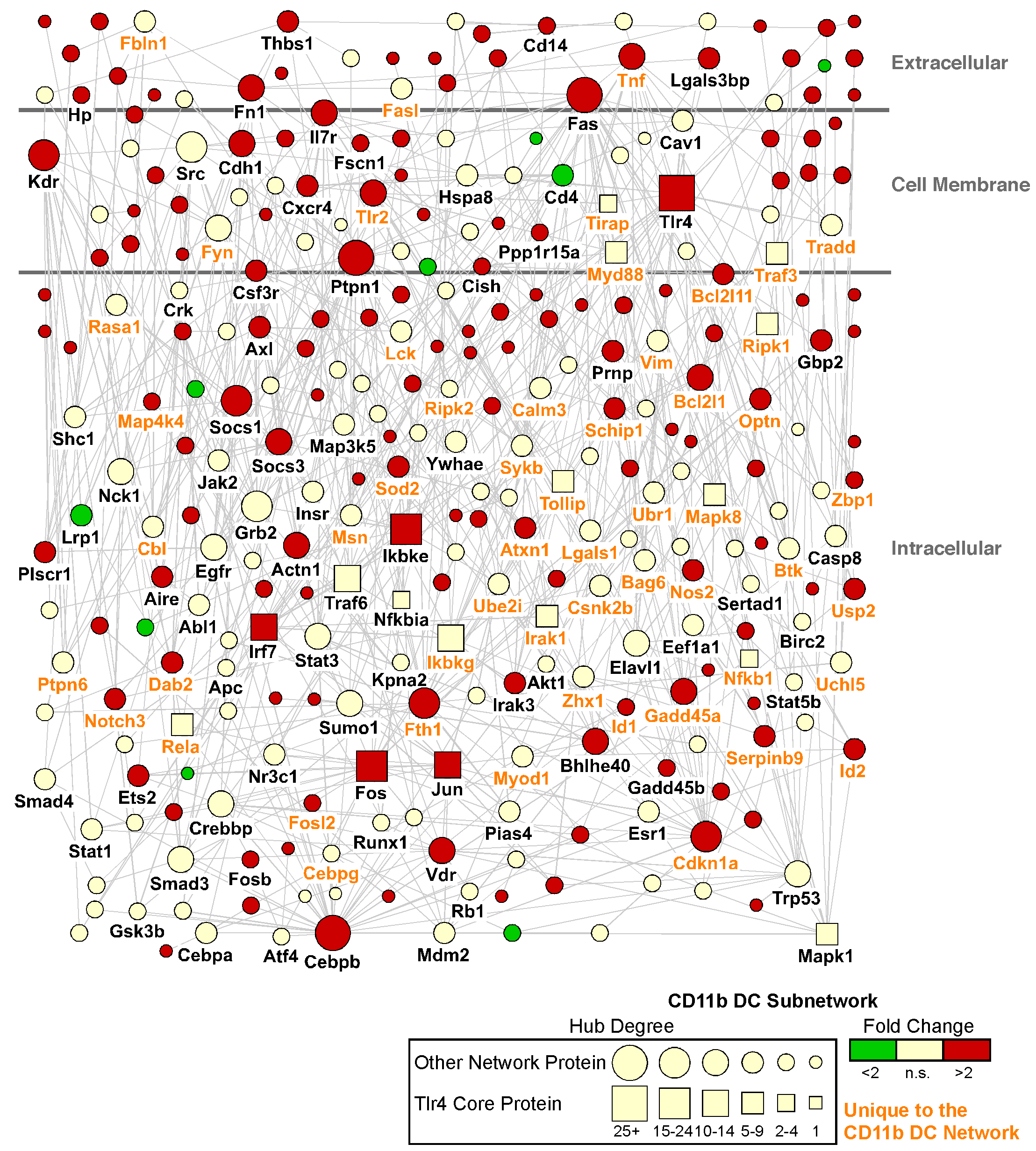

Supplement: Figure S4 — Network analysis of LPS-responsive genes in CD11b DCs. A network analysis was carried out on the transcriptional response of CD11b DCs stimulated in vivo with LPS as compared to steady-state. Subnetwork analysis was used to enrich networks in an unbiased manner for interactions with differentially expressed genes. The figure was made using the Cytoscape plugin Cerebral to show the cellular localisation of each gene. Node size is proportional to its Hub degree (interconnectivity with other genes/nodes), and node colour indicates relative gene expression (+LPS/−LPS). Square nodes represent core LPS response molecules. Nodes labelled in orange text are present in the CD11b but not CD8 DC subnetwork, while nodes labelled in black text are present in both. Networks were organised using the Cytoscape plugin Cerebral, which organises nodes based on their relative cellular location. For visualisation, only selected nodes are labelled. The full list of nodes/network characteristics is provided in Table S6. (TIFF) [file pone.0100613.s004.tif]

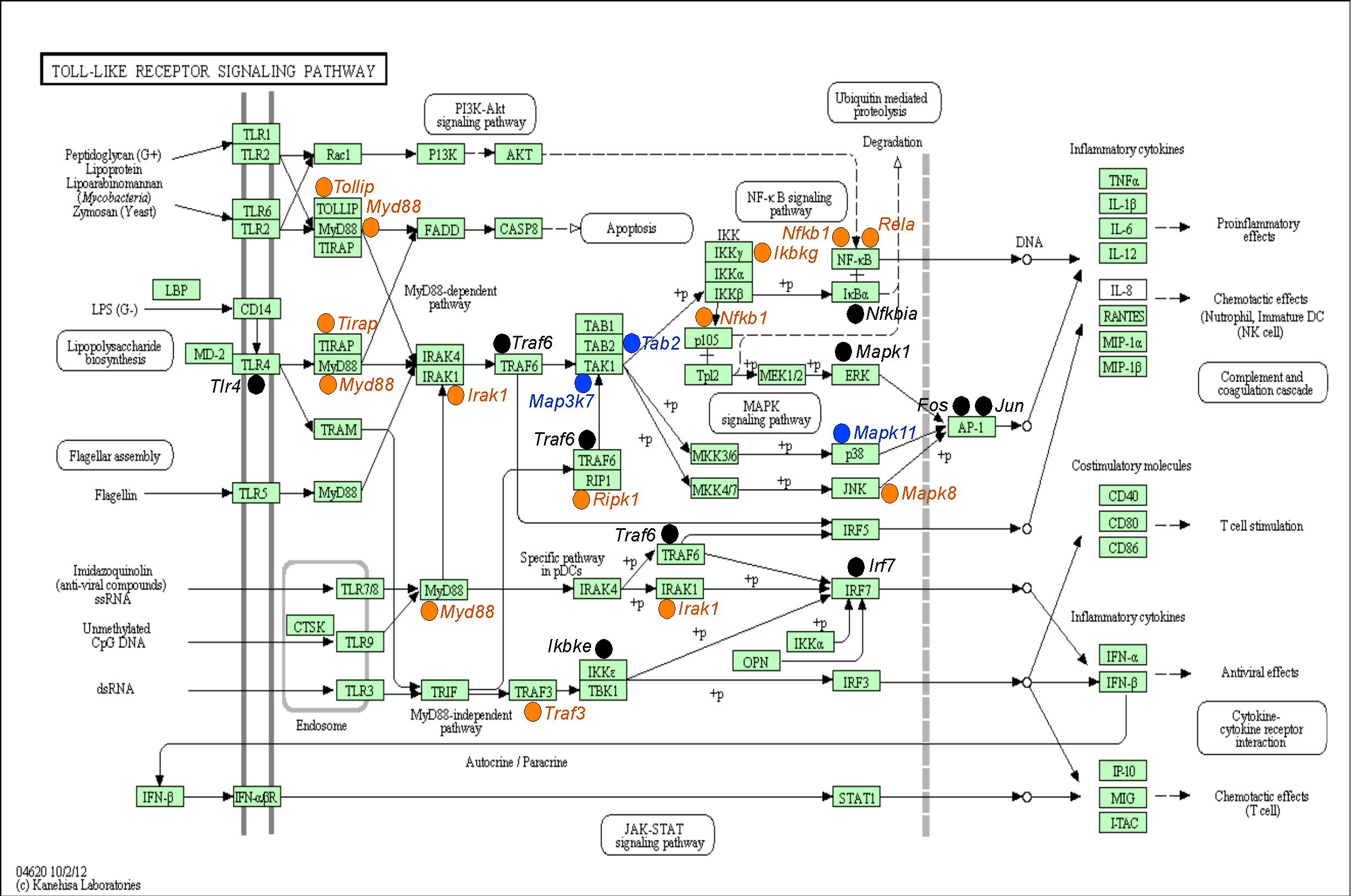

Supplement: Figure S5 — Subset-specific Hubs in relation to a KEGG pathway map of TLR signalling. Core LPS response Hubs identified in the subnetwork analysis of CD8 or CD11b are identified by coloured dots and gene names (italics) overlayed on a KEGG pathway map. Black dots and text indicate Hubs identified in both subnetworks, blue indicates Hubs identified only in the CD8 subnetwork and orange indicates Hubs identified only in the CD11b subnetwork. (TIFF) [file pone.0100613.s005.tif]
