# Supplementary material for: A Systems Biology Approach to the Analysis of Subset-Specific Responses to Lipopolysaccharide in Dendritic Cells
Source: PLoS One. 2014 Jun 20;9(6):e100613. doi: 10.1371/journal.pone.0100613 (PMC4065045; doi:10.1371/journal.pone.0100613)
Supplement: Table S1 — List of reanalysed datasets and their associated references. (DOCX) [file pone.0100613.s006.docx]

**Table S1.** List of reanalysed datasets and associated references.

| **Dataset number** | **Dataset reference code** | **Meta-Analysis** | **Experiment** | **Notes** | **Refs** |
| --- | --- | --- | --- | --- | --- |
| 1 | GSE339 | Spleen DCs |  |  | [[16](#_ENREF_16)] |
| 2 | GSE9810 | Spleen DCs |  |  | [[17](#_ENREF_17)] |
| 3 | GSE30022 | Spleen DCs |  |  | NA |
| 4 | GSE15907 | Spleen DCs |  | Analysed from raw data | [[18-21](#_ENREF_18)] |
| 5 | GSE12392 | Spleen DCs |  |  | [[22](#_ENREF_22)] |
| 6 | GSE29949 | Spleen DCs |  |  | [[23](#_ENREF_23)] |
| 7 | GSE6259 | Spleen DCs | -Selected WT DCs  -Not Ftl3L-stimulated DCs |  | [[24](#_ENREF_24)] |
| 8 | GSE35458 | Spleen DCs |  |  | [[25](#_ENREF_25)] |
| 9 | GSE10246 | Spleen DCs |  |  | [[26](#_ENREF_26)] |
| 10 | GSE28340 | LPS BM-DCs | -GM-CSF+IL-4 for 7d  -CD11c+ selection  -500ng/ul LPS for 12hrs |  | [[10](#_ENREF_10)] |
| 11 | GSE36009 | LPS BM-DCs | -GM-CSF for 8d  -1ug/ml LPS O/N |  | [[11](#_ENREF_11)] |
| 12 | GSE32381 | LPS BM-DCs | -GM-CSF+SCF for 4d, GM-CSF 4d  -100ng/ml LPS for 4h | Analysed from raw data | [[12](#_ENREF_12)] |
| 13 | GSE17721 | LPS BM-DCs | -GM-CSF for 5d  -CD11c+ selection  -100ng/ml LPS for 4, 6, 8, 12, 16, and 24h | The 0.5, 1, and 2hr timepoints were excluded (too few DE genes) | [[13](#_ENREF_13)] |
| 14 | GSE15087 | LPS BM-DCs | -GM-CSF 7d  -1ug/ml LPS for 6h |  | [[14](#_ENREF_14)] |
| 15 | GSE7219 | CD11c+ cDCs | -25ug LPS and 100ug anti-CD40 antibody i.p.  -DC isolated 6 hours after treatment |  | [[15](#_ENREF_15)] |
| 16 | GSE10246 | Macrophages | -LPS for 6-7h | Thio vs. BM-M | [[26](#_ENREF_26)] |
| 17 | GSE3720 | γδ T cells | -10mg/ml LPS for 4h | Vδ1 vs. Vδ2 | [[38](#_ENREF_38)] |
| 18 | GSE7850 | Endothelial cells | -100ng/ml LPS for 4h | Choroidal vs. Retinal Vascular | [[39](#_ENREF_39)] |
| 19 | GSE39840 | Cord-Blood | -10ng/ml LPS for 4h | Neutrophils vs. Monocytes | [[46](#_ENREF_46)] |

DCs: dendritic cells; BM-DCs: Bone marrow-derived DCs; BM-M: Bone marrow-derived macrophages; Thio: Thioglycolate-elicited peritoneal macrophages
